# Interventions to increase vaccination in vulnerable groups: rapid overview of reviews

**DOI:** 10.1186/s12889-024-18713-5

**Published:** 2024-06-03

**Authors:** Gill Norman, Maartje Kletter, Jo Dumville

**Affiliations:** 1 NIHR Innovation Observatory, Population Health Sciences Institute, Newcastle University, Newcastle upon Tyne, UK; 2 Evidence Synthesis Group, Population Health Sciences Institute, Newcastle University, Newcastle upon Tyne, UK; 3 Division of Nursing, Midwifery & Social Work, School of Health Sciences, Faculty of Biology, Medicine and Health, University of Manchester, Manchester, UK

**Keywords:** Vaccination, Inequity, Vulnerable groups, Minorities, Intervention, Systematic review

## Abstract

**Objective:**

Groups which are marginalised, disadvantaged or otherwise vulnerable have lower uptake of vaccinations. This differential has been amplified in COVID-19 vaccination compared to (e.g.) influenza vaccination. This overview assessed the effectiveness of interventions to increase vaccination in underserved, minority or vulnerable groups.

**Methods:**

In November 2022 we searched four databases for systematic reviews that included RCTs evaluating any intervention to increase vaccination in underserved, minority or vulnerable groups; our primary outcome was vaccination. We used rapid review methods to screen, extract data and assess risk of bias in identified reviews. We undertook narrative synthesis using an approach modified from SWiM guidance. We categorised interventions as being high, medium or low intensity, and as targeting vaccine demand, access, or providers.

**Results:**

We included 23 systematic reviews, including studies in high and low or middle income countries, focused on children, adolescents and adults. Groups were vulnerable based on socioeconomic status, minority ethnicity, migrant/refugee status, age, location or LGBTQ identity. Pregnancy/maternity sometimes intersected with vulnerabilities. Evidence supported interventions including: home visits to communicate/educate and to vaccinate, and facilitator visits to practices (high intensity); telephone calls to communicate/educate, remind/book appointments (medium intensity); letters, postcards or text messages to communicate/educate, remind/book appointments and reminder/recall interventions for practices (low intensity). Many studies used multiple interventions or components.

**Conclusion:**

There was considerable evidence supporting the effectiveness of communication in person, by phone or in writing to increase vaccination. Both high and low intensity interventions targeting providers showed effectiveness. Limited evidence assessed additional clinics or targeted services for increasing access; only home visits had higher confidence evidence showing effectiveness. There was no evidence for interventions for some communities, such as religious minorities which may intersect with gaps in evidence for additional services. None of the evidence related to COVID-19 vaccination where inequalities of outcome are exacerbated.

**Prospero registration:**

CRD42021293355

**Supplementary Information:**

The online version contains supplementary material available at 10.1186/s12889-024-18713-5.

**Key points**

- Inequity in vaccination is a recognised public health issue which has been amplified by the COVID-19 pandemic.
- This overview uses rapid but rigorous methods to systematically review evidence from over 20 systematic reviews of interventions for increasing vaccination in marginalised, disadvantaged or otherwise vulnerable groups.
- We identify and evaluate evidence for low, medium and high intensity interventions targeting pull factors (increasing demand for vaccination); push factors (increasing access to vaccination); and vaccination providers.
- We highlight the gaps in evidence for key interventions to improve access, for COVID-19 vaccination and for groups such as religious minorities.

## Background

Comparatively low vaccination rates in underserved groups are a recognised UK public health issue. Impacted groups include those who are socio-economically disadvantaged, people from black and minority ethnic backgrounds, and other groups who are marginalised, disadvantaged or otherwise vulnerable [1]. 

These known issues in vaccination inequalities were amplified during the COVID-19 pandemic. There is recent evidence that inequities in COVID-19 vaccination rates are even greater than in influenza vaccination, where vulnerability to disease may be similarly distributed in older people and those with pre-existing conditions. For example, in communities across Greater Manchester, a city-region with approximately 2.8 million people and considerable population diversity, inequality of vaccination relative to white British residents was greater for all except one of 16 minority ethnic groups for the first dose of COVID-19 vaccine than it was for influenza vaccination [2]. 

People from minoritised and disadvantaged groups are disproportionately likely to experience negative outcomes from COVID-19 infection, including hospitalisation, intensive care unit admission, and death [3]. Given that inequality of vaccination is disproportionately concentrated among those at greatest risk from the disease, the need for interventions which can address vaccination inequity is particularly acute in the context of COVID-19 vaccination campaigns, including annual booster campaigns for older or clinically vulnerable people. Learning, however, is also relevant to wider vaccination campaigns.

A 2015 overview of reviews identified 15 systematic reviews of strategies for so-called vaccine hesitancy, but few interventions which specifically targeted those who were labelled vaccine hesitant [4]. Most of the included reviews also focused on childhood vaccination campaigns. This review is now substantially out of date, particularly in the context of the COVID-19 pandemic, and the exacerbation of vaccination inequity seen in its early stages. Our preparatory work highlighted further relevant literature and reinforced the need for a new systematic overview of reviews focused on interventions to reduce vaccination inequalities in underserved groups.

### Objectives

This rapid overview of reviews was undertaken to identify and assess the evidence for effectiveness of interventions to increase vaccination in underserved, minority or vulnerable groups.

## Methods

The protocol for this overview of reviews was registered on Prospero: CRD42021293355 [5]. We adapted appropriate rapid systematic review methods for this rapid overview and reported it following PRIOR reporting guidelines where possible [6]. 

### Inclusion criteria

We included systematic reviews which contained randomised controlled trials (RCTs) of interventions for increasing vaccination in groups of people who were considered to be underserved, minoritised or otherwise vulnerable in the context of the vaccination activities investigated. We did not otherwise limit eligibility and accepted authors’ definitions of these groups. We did however consider older age to be a source of vulnerability as well as groups which may be marginalised based on ethnicity, socioeconomic status, place of residence, faith, or LGBTQ + identity. We treated pregnancy/maternity as an additional vulnerability but not itself as a sufficient reason to consider populations vulnerable (so we included interventions targeted at pregnant women/new mothers eligible for other reasons, but not interventions for all pregnant women or families with young children).

Systematic reviews were defined as reviews which included as a minimum: a systematic search; specific inclusion criteria; and an identifiable set of included studies. We only included English-language reviews; reviews in other languages would have been noted but not extracted. We included reviews that contained RCTs evaluating any intervention aimed at increasing vaccination rates in groups of interest, even if reviews were not exclusively aimed at these groups. Interventions could be delivered in any clinical or community setting and in any country, although we considered the relevance of settings in our synthesis. We included any comparator including alternative interventions, no intervention, or provision of usual healthcare/standard vaccination campaigns.

Our primary outcome was vaccination, broadly defined as we anticipated a range of reported measures. In the absence of evidence for vaccinations we would have considered measures such as willingness/intention to vaccinate and knowledge about vaccinations.

### Search

We searched the Cochrane Database of Systematic Reviews, Ovid Medline, Ovid Embase and Ebsco CINAHL from inception to 25 November 2022 (updating an initial search in December 2021) without language or date restrictions. For search strategies see supplementary information (Appendix 1). We also checked references of included studies. Search results were deduplicated using Endnote X20 [7]. 

### Selection of studies

We used Rayyan to screen search records [8]. To increase rapidity, 10% of titles and abstracts were screened in duplicate by two independent researchers for calibration and consistency. Remaining citations were single screened with a second researcher consulted in cases of uncertainty; disagreements were resolved through discussion. Full texts were obtained for all potentially eligible studies. After initial single screening of these full texts, all reviews which were not clearly included or excluded were screened by a second independent researcher; because of the nuanced nature of the inclusion criteria in relation to vulnerable groups this was most reviews. All relevant reviews were included; overlap in included studies was managed post-inclusion.

### Data extraction and assessment of risk of bias

We piloted a bespoke Microsoft Excel data extraction form on a small sample of reviews. After this one researcher extracted the data and a second was consulted in cases of uncertainty. Extraction focused on review and study level data and key review findings. Some reviews contained only a portion of studies eligible for our overview e.g. some included reviews contained RCTs and non-RCTs or RCTs assessing irrelevant interventions or populations. In these cases, only relevant data were extracted. We extracted the following: number and size of relevant RCTs and their intervention characteristics; vaccination types; participants and vulnerabilities; outcome data; results of quality appraisal, risk of bias and/or GRADE assessment (Grading of Recommendations Assessment, Development and Evaluation) [9]. 

We assessed risk of bias in reviews using ROBIS; one researcher performed the assessments and a second checked these [10]. 

### Data synthesis

We followed recommendations of the Synthesis Without Metanalysis (SWiM) approach in the synthesis of data, adapted to our rapid overview of reviews [11]. We developed the following framework to support narrative synthesis of finding, (this is an expansion and codification of the approach planned in the protocol).

We focused on the primary outcome of vaccination (including vaccination, completion of vaccination schedules, and being up to date with vaccinations); outcomes such as willingness/intention to vaccinate or knowledge about vaccination were considered indirectly relevant.

We first adapted the approach of Ward 2012 [12] and grouped interventions into three sets based on type and purpose of the intervention: interventions to increase demand for vaccination (targeting pull factors); interventions to increase access to vaccination (targeting push factors); and interventions targeting vaccination providers. We considered that interventions which were primarily provider-focused would also fall into the other two categories for their impact on patients but considered that the provider focus was important to consider separately. Within these sets of interventions we then followed Thomas 2018 [13], and considered the intensity of the intervention delivery as: high intensity (e.g. home visits); medium intensity (e.g. telephone calls); or low intensity (e.g. text messages). This intensity categorisation was based on resource requirements for providers rather than possible patient perception re the intensity of receiving the intervention. When interventions were multi-component or multi-level we noted this. Two researchers agreed on groupings by intervention purpose and intensity and resolved disagreements through discussion.

We mapped RCT overlap between reviews using GROOVE and paid particular attention to this issue of overlapping primary studies for interventions where contributing reviews showed overlap, in order to reduce the risk of double weighting data [14]. 

We considered differences in findings between the countries where studies were undertaken, particularly noting whether the studies were undertaken in high income countries or in low or middle income countries (LMIC). This included consideration of the specific populations and groups targeted.

### Assessing confidence in synthesised findings

We assessed confidence in findings using a GRADE-informed approach [9]. One researcher made judgements and consulted a second in cases of uncertainty. We made initial judgements for each intervention in each review then considered evidence across the overview, taking into account overlapping data [14]. We assigned greatest confidence to interventions where there was consistent evidence for effectiveness from reviews with low risk of bias, which provided either GRADE assessment or reported evidence from larger RCTs that were described in reviews as well-conducted with clearly reported effect estimates. There is necessarily more subjectivity and estimation in these judgements than in GRADE because of often incomplete information; a formal GRADE judgement would overstate our certainty about evidence quality [9]. We have used the terms “higher, medium and lower confidence” to denote these judgements.

## Results

### Results of the search

**Fig. 1:**
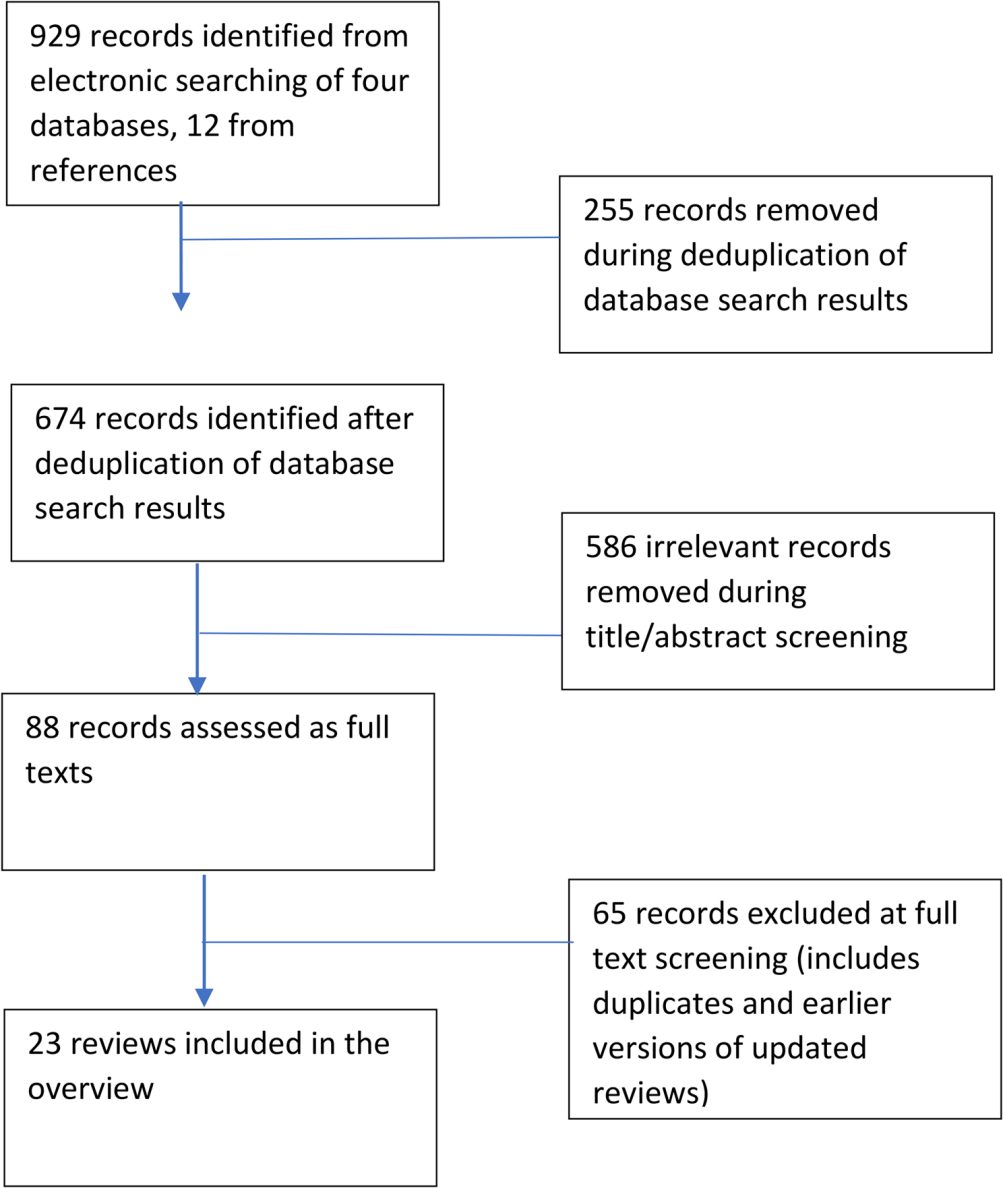
Flow diagram for records identified for the review

We identified 674 records following deduplication and assessed 88 full texts. We included 23 reviews [13, 15–36]. (Fig. 1). Sixty-five full texts were excluded for the following reasons: not a systematic review; a review protocol; an earlier version of a Cochrane review; not relevant to interventions to improve vaccination-related outcomes; did not include any relevant RCTs. An excluded studies list is available on request.

### Characteristics of included reviews

Characteristics of included reviews are summarised in Tables 1 and 2. Reviews were published between 1998 and 2022; most were recent with nine published in 2021 or 2022 and 17 since 2017. Fifteen reviews included studies from high income countries and eighteen included either adults or adolescents (Table 1). Eleven reviews looked at multiple types of vaccination (of which five focused on childhood vaccinations); six reviews looked at influenza vaccination, five at HPV vaccination and one at hepatitis B vaccination (Table 2).

**Table 1:** Summary of review characteristics

Populations & countries	High income	Low/Middle income	Mixed/unclear	Total
Children only	1	1	3	5
Adolescents only	1	-	1	2
Adults only	5	-	-	5
Mixed/unclear	8	1	2	11
Total	15	2	6	23

**Table 2:** Characteristics of the included reviews

Review ID	Setting	Age	Vaccinations	Vulnerability type	Included studies (relevant RCTs)	Intervention type	Overall risk of bias in review (ROBIS)
Balzarini 2020 [15]	High income	Adults only	Multiple	Age	8 (5)	Electronic health records	Low
Brandt 2021 [16]	High income	Adolescents only	HPV	Location	15 (7)	Multiple	High
Callahan 2021 [17]	High income	Adults only	Influenza	Ethnic minority Pregnancy/maternity	12 (4)	Multiple	High
Crocker-Buque 2017a [19]	Low/middle income	Mixed	Multiple	Socioeconomic Location	63 (4)	Multiple	High
Crocker-Buque 2017b [18]	High income	Mixed	Multiple	Ethnic minority Location Socioeconomic	41 (17)	Multiple	High
Glenton 2011 [20]	High and low/middle income	Children only	Childhood immunisations	Socioeconomic	12 (10)	Lay health workers	Low
Gopalani 2022 [21]	High income	Mixed	HPV	Ethnic minority (first nations)	15 (1)	Educational	Unclear
Isenor 2016 [22]	High income	Adults only	Multiple	Age Socioeconomic	36 (1)	Pharmacists	Low
Kaufman 2018 [23]	High and low/middle income	Children only	Childhood vaccination	Pregnancy/maternity Socioeconomic Age	10 (2)	Face-to-face interventions	Low
Kendrick 2000 [24]	High and low/middle income	Children only	Childhood vaccination	Ethnic minority Socioeconomic Location Other vulnerability	11 (9)	Home visiting programmes	Unclear
Lambert 2021 [25]	High income	Mixed	Respiratory-related childhood diseases	Migrant/refugee status	9 (1)	Multiple	Unclear
Lewin 2010 [26]	High and low/middle income	Mixed	Multiple	Socioeconomic	82 (8)	Lay health workers	Low
Lott 2020 [27]	High income	Mixed	HPV	Ethnic minority Age LGBTQ	9 (8)	Multiple	Unclear
Machado 2021 [28]	High income	Children only	Childhood vaccination	Socioeconomic	40 (17)	Multiple	Low
Mogaka 2019 [29]	Unclear	Adolescents only	HPV	Socioeconomic Ethnic minority Location Other vulnerability	11 (3)	Educational	High
Mohammed 2021 [30]	High income	Mixed	Influenza	Age Ethnic minority Pregnancy/maternity	52 (≥ 1)^1^	Multiple	High
Murray 2021 [31]	High income	Adults only	Influenza	Age	12 (2)	Pharmacists	Low
Nelson 2016 [32]	Low/middle income	Children only	Childhood vaccination	Location Socioeconomic	15 (6)	Multiple	Unclear
Odone 2015 [33]	High income	Mixed	Multiple	Age Ethnic minority	19 (6)	mHealth	Unclear
Rani 2022 [34]	High income	Mixed	HPV	Ethnic minority	30 (5)	Public education	High
Sarnoff 1998 [35]	High income	Adults only	Influenza	Age Other vulnerability	16 (10)	Multiple	High
Thomas 2018 [13]	High and low/middle income	Adults only	Influenza	Age	61 (61)	Multiple	Low
Vedio 2017 [36]	High income	Unclear	Hepatitis B	Ethnic minority Migrant/refugee status	48 (1)	Multiple	Low

^1^It was unclear how many relevant RCTs were included in the review. Attempts to contact authors for clarification were unsuccessful

### Underserved groups represented included

- Low socioeconomic status (9 reviews).
- Ethnic minority or first nations people (10 reviews).
- Migrant or refugee status (2 reviews).
- Age (9 reviews).
- Location (6 reviews).
- LGBTQ identity (1 review).
- Other (6 reviews).

These vulnerabilities co-occurred in many studies; in three reviews socioeconomic status, age or ethnic minority status co-occurred with the additional vulnerability of pregnancy/maternity (Table 2). We did not identify any reviews with RCTs targeting faith groups.

### Risk of bias

**Fig. 2:**
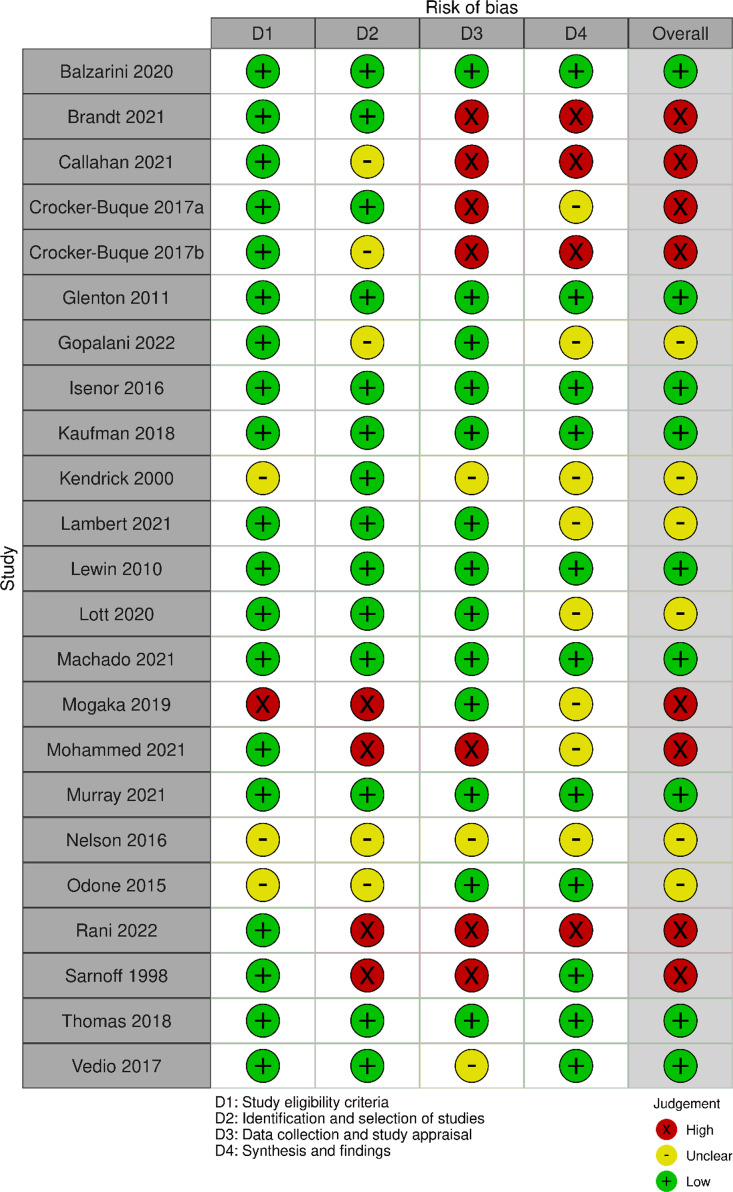
Summary of ROBIS assessments for included reviews

The ROBIS assessment found that nine reviews had low, six unclear, and eight high overall risk of bias (Fig. 2; Table 2). Full responses to signalling questions are available on request.

### Overlap

**Fig. 3:**
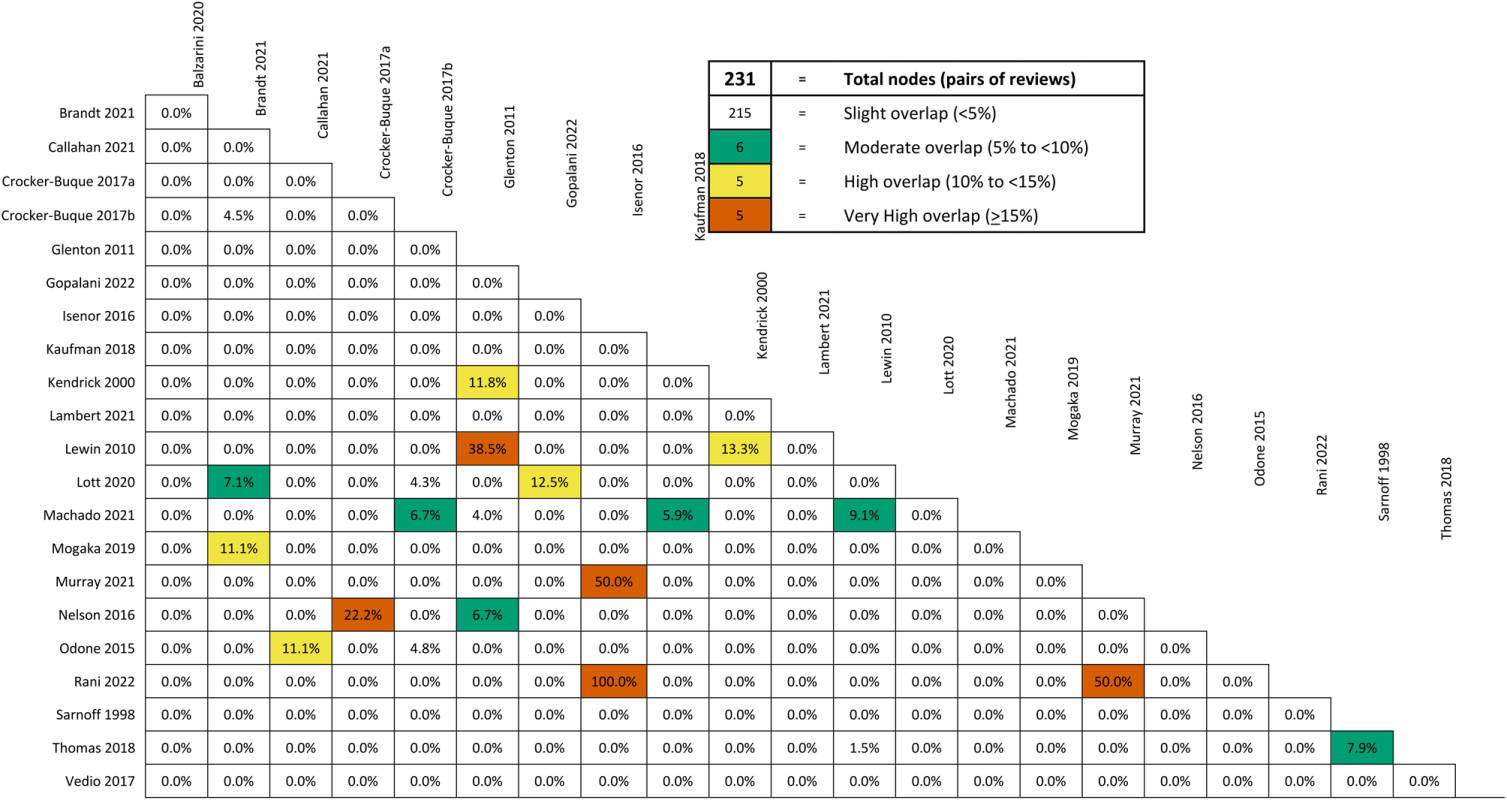
Summary of GROOVE assessment of overlapping RCTs in included review

Mapping of included RCTs using GROOVE identified 16 pairs of reviews with moderate (six), high (five) or very high (five) overlap (Fig. 3) [14]. Nineteen individual reviews contributed to the overlap; three reviews overlapped at least moderately with three other reviews and seven with two other reviews. Nine reviews [16, 19–21, 24, 26, 27, 29, 32] were linked through the three reviews with the highest number of overlaps [20, 24, 26]. Because overlap was substantive we were careful to consider evidence from individual RCTs, and pay particular attention to overlap for interventions in reviews with highest overlap, which included home visiting and various educational and communication interventions.

### Effectiveness of interventions

Unless otherwise stated, all interventions are compared with usual care, the outcome is vaccination, and effects favour interventions. Where we have medium rather than higher confidence this is because of combinations of concerns around one or more of: reporting, study quality, inconsistency of results or limited numbers of participants. We have reported reviews’ GRADE assessments where these were available. Full documentation of evidence for interventions is in Supplementary Information (Appendix 2). Below we summarise narratively interventions for which we have higher or medium confidence, grouped by intervention intensity, purpose and type. For interventions where we have lower confidence see Tables 3 and 4. Table 3 summarises all interventions where evidence identified a benefit of the intervention; Table 4 summarises those interventions where current evidence did not identify a benefit.

**Table 3:** Summary table of interventions with some evidence of effectiveness

Intensity	Increase demand	Increase access	Provider-focused
High	**Home visits** Home visits for communication/education^a^ Home visits to communicate as highest intensity^a^ component of escalating intervention ^a^ Educational message in home visits by medical students^b^ **Advocacy** Pharmacists or community volunteers advocating for vaccination^b^ Community lay educators ^b^ **Education** Short educational sessions^b^ Information in ESL classes^b^	**Home visits** Home visits to vaccinate^a^ **Additional/different services or staff** Additional clinics as part of a multicomponent intervention^b^ Pharmacist initiated vaccination programmes^b^ Delivery of vaccination by lay healthcare workers^c^ **Partnership and outreach** Community partnership as part of a multicomponent intervention^b^ Routine or general clinic visits used to vaccinate as part of a multicomponent intervention^b^	**Facilitators** Facilitator visits to health care practices (range of interventions delivered)^a^ **Education** Educational sessions for providers^c^
Medium	**Telephone calls to communicate or educate** Telephone reminders to attend or book appointments^a^ Telephone calls from lay healthcare workers ^a^ Telephone calls as part of multicomponent ^a^ intervention (targeted calls or intermediate stage of escalating intervention) ^a^ Telephone calls to adolescents plus parents versus parents only ^a^ Telephone education as part of multicomponent intervention ^c^	**Routine/general clinic appointments to vaccinate** Using routine visits to clinics or healthcare providers to vaccinate as part of a multicomponent intervention ^b^	**Case management** Case management as part of multicomponent intervention ^b^
Low	**Postal communication to communicate or educate** Letters or postcards to communicate/educate ^a^ Letters or postcard reminders to attend or book appointments ^a^ Personalised letters versus generic ones ^b^ Letters in appropriate community language ^b^ Postcards using accessible language ^b^ **Emails to communicate or educate** Individually tailored emails ^b^ **Text messages to communicate/educate** Text messages to communicate/educate ^a^ Single or multiple text message reminders to attend or book appointments ^a^ Text and postal communications s as part of multicomponent interventions ^b^ Text message appointment reminders versus postal reminders ^b^ Text messages with educational component or interactivity versus other text messages ^c^ **Written material given in person** Pamphlets given in person ^b^ Redesigned immunisation cards ^b^ Pictorial information used at home visit ^b^ Distributing information with promotional T-shirts ^c^ Printed educational material including flyers ^c^ **Mass media** Use of mass media including in community language ^b^ **Video material** Home-delivered DVDs ^c^		**Use of technology systems** Personalised electronic health records^a^ Centralised reminder/recall systems for vaccination instead of local practice-based systems^a^ Computerised reminders to vaccinate^b^ **Prompts, orders or instructions to vaccinate** Reminders to physicians to vaccinate^a^ **Information or incentives** Academic detailing or benchmarking physicians (peer comparisons) including clinic posters presenting vaccination rates ^b^ Payments to physicians^b^

a = high confidence in evidence; b = medium confidence in evidence; c = lower confidence in evidence

**Table 4:** Summary table of interventions where there is no current clear evidence of effectiveness

Intensity	Increase demand		Increase access		Provider-focused			
High		**Home visits** Home visits as part of an enhanced perinatal care programme ^b^ Enhanced perinatal visits compared to standard visits ^b^ **Alternative arrangements for support** Group versus individual well child sessions ^c^ **School interventions** School engagement ^c^ School science education versus brochure ^c^ Computer based interventions in schools ^c^	**Interventions to increase accessibility of services** Health service navigation ^c^ Placing vaccinator at front of clinic ^c^ Vaccine champions as part of practice-based initiative ^c^ Patient-provider talking points ^c^		**Outreach, reminders and feedback** Educational reminders, academic detailing and peer comparisons versus mailed educational materials ^a^ Educational outreach plus written feedback versus written feedback ^a^ **Education** Education sessions for providers ^c^			
Medium	**Phone calls** Phone call plus pharmacist vaccine delivery versus pharmacist vaccine delivery only ^c^				**Reminders** Prompts to healthcare workers to vaccinate as a single intervention ^c^			
Low	**Video and/or online material** Use of videos in clinic or public spaces ^c^ Culturally appropriate online storytelling ^c^ Online bilingual education videos ^c^				**Communication** Posters plus postcards versus posters alone ^a^			

a = high confidence in evidence; b = medium confidence in evidence; c = lower confidence in evidence

### High-intensity interventions: increasing demand for vaccination

#### Home visits for communication or education

We have higher confidence that home visits for the purposes of communication by health professionals, lay health workers, volunteers and students increase vaccination in underserved groups (11 reviews) [13, 18–20, 23–26, 28, 32, 35]. We drew primarily on evidence from Cochrane reviews finding moderate certainty evidence in, respectively, influenza vaccinations for older adults; [13] and childhood vaccinations in economically disadvantaged families being visited by lay healthcare workers; [26] the evidence was broadly consistent across the other reviews, which included a variety of vulnerable groups. Home visits were also the highest intensity component of interventions using escalating intensity of reminders, and were used in multicomponent interventions; both were effective for disadvantaged children and adolescents. Evidence for home visits compared with postal reminders was inconsistent [20]. 

#### Advocacy

We have medium confidence that community volunteers or pharmacists advocating for vaccination may increase vaccination in some underserved groups [31, 32]. Each review contained a single relevant medium-sized or large RCT in different groups (older adults and children in urban/disadvantaged groups respectively).

#### Community partnership and outreach

We have medium confidence in the effectiveness of community partnership and outreach within multicomponent interventions (four reviews). This includes outreach as part of a multicomponent intervention; [28] lay health workers leading focus groups (groups cascade information to the community; moderate certainty evidence); [20] and community involvement in motivating vaccine acceptance [32], and ensuring relevance of reminders [18]. 

#### School and other non-home-based in person educational interventions

Eight reviews contributed evidence for varied interventions [16, 18, 19, 21, 29, 32, 34, 36]. We have medium or lower confidence in this evidence. Reviews found school-based interventions do not currently have clear evidence of effectiveness in impacting vaccination in underserved groups [16, 18]. Educational sessions delivered to adults in a range of settings including English as a second language (ESL) classes [36] and community venues showed mixed results, with some RCTs reporting benefits. Interventions in LMIC contexts found positive effects from interventions targeting parents (pictorial information or redesigned vaccination cards alongside a verbal educational message) [19, 32], or brief interventions for adolescents [29]. Evidence from high income contexts primarily related to HPV vaccination for adolescent girls in the US and mostly did not show evidence of an effect [16, 18, 21, 34]. RCTs in two reviews found benefits in increasing vaccination via interventions for mother-daughter dyads in minority ethnic communities [21, 34]. 

### High intensity interventions: increasing access to vaccination

#### Home visits for vaccination

We have higher confidence that vaccination during home visits (delivered by health care professionals, students or community healthcare workers) increases vaccination compared to standard care (invitations to clinic) [13, 19, 32]. Most evidence comes from the Cochrane review in influenza vaccination for older adults, (high certainty GRADE assessment based on two RCTs) [13], but there is also evidence from childhood vaccination in LMIC.

#### Using different/additional locations or services or staff to deliver vaccinations

We have medium confidence in effectiveness of additional clinics as part of a “four pillars” multicomponent intervention [18]. We found no evidence for them as standalone interventions. We also have medium confidence in using routine/general clinic visits to vaccinate within a multilevel, multicomponent intervention [28]. For both interventions our confidence is reduced because the impact of the availability component cannot be isolated. Using group visits of participants to clinics may also be effective (moderate certainty evidence from a Cochrane review including one RCT) [13]. We have medium confidence in pharmacist-initiated vaccination programmes involving use of pharmacy-based services and/or delivery by pharmacy staff [22, 31, 35]. 

### Provider focused interventions

#### Facilitators for healthcare professionals

We have higher confidence that facilitator involvement with healthcare practices increases vaccination; this is based on moderate certainty evidence from a Cochrane review of influenza vaccination for older adults [13]. . Two cluster RCTs targeted several goals using multiple strategies over 12 to 18 months, including practice visits by facilitators and approaches such as baseline audit, ongoing feedback, consensus building, and follow-up. One study also used educational materials for professionals and patients. Both these studies showed increased numbers of eligible older people vaccinated, a smaller study of a facilitated educational group plus audit did not find a clear effect [13]. 

### Medium-intensity interventions: increasing demand for vaccination

#### Telephone calls for communication and education

We have higher confidence that the following increase vaccination: telephone calls to remind people about booked appointments, deliver reminders about booking appointments, and provide information about vaccination processes (six reviews) [13, 16, 18, 26, 28, 35]. We have medium confidence in the effectiveness of using phone calls to adolescents as well as their parents; and for telephone calls as part of multicomponent interventions [16, 18, 28], including targeted phone calls and phone calls in an intervention with escalating intensity of reminders [18]. 

### Medium-intensity interventions: increasing access to vaccination

Included reviews did not report on medium intensity interventions primarily aiming to increase access to vaccination; however some provider-focused interventions (e.g. changing provider systems to allow use of routine visits for vaccination) are likely to have increased access to vaccination.

### Medium-intensity interventions: provider-focused interventions

#### Case management

We have medium confidence in case management within a multicomponent intervention: this included feedback on missed opportunities to vaccinate, tracking, triage and flagging of vaccination status [18, 28]. 

#### Routine visits

Using routine visits to vaccinate: We have medium confidence in using routine visits to healthcare providers or clinics to vaccinate as part of a multicomponent intervention for childhood vaccinations [28], and lower confidence as a stand-alone intervention in older adults [35]. 

### Low-intensity interventions: increasing demand for vaccination

Various methods involving written material for communication and education were used. Both texts and postal communications were used as stand-alone interventions and as part of multicomponent or escalating intensity interventions, where it is harder to determine their impact [18, 26]. These multicomponent or escalating interventions showed evidence of effectiveness; we summarise evidence for different delivery methods here.

#### Text messages

We have higher confidence that single or multiple text message reminders to attend or book appointments (multiple trials in seven reviews) increase vaccination [16–19, 27, 28, 33]. Text messages for appointment reminders may be more effective than postal communication [18]. Unlike phone calls and postal communication there was limited evidence for an impact of text messages in older populations, where risk of digital exclusion may be higher. Evidence for effectiveness of different types of text messages such as using different messages or interactivity is limited (Table 4).

#### Emails, online messages, mass media and videos

We have medium confidence that individually tailored reminder emails are effective [27], although there is less evidence for email and online messages than other forms of written messaging, with most examples being elements of wider communication strategies [16, 27, 28]. We only had lower confidence in assessments of video-based messaging [16, 17, 27, 29, 34], online messages [27], and mass media [29, 36]. In each case the evidence comprised single studies with limitations of reporting, power and study methods.

#### Postal communication reminders

We have higher confidence that postal reminders to attend or book appointments, and for providing information about vaccination are effective (six reviews). Postcards and letters were the most used; [13, 16, 18, 26, 28, 30] postcards were identified as particularly effective [13]. Personalised letters may be more effective than generic ones [28]. We have medium confidence in evidence for sending letters in an appropriate community language (e.g. Spanish for US Hispanic communities) [27], and for use of postcards designed to use (accessible) “universal language” [18]. 

#### Written material given in person

We have medium confidence that brief paper-based information given in person increase vaccination (three reviews). This included redesigned immunisation cards with the next appointment date in large print, with or without a short verbal intervention; use of pictorial information cards as an additional intervention during home visits; [19, 32] and providing a pamphlet of information with or without a short verbal intervention [17]. 

### Low intensity interventions: increasing access to vaccination

Included reviews did not report on low intensity interventions aiming to increase access to vaccination.

### Low intensity interventions: provider-focused interventions

#### Centralised systems

We have higher confidence in the effectiveness of centralised reminder/recall systems compared to practice-based reminder/recall systems in increasing the number of children up to date on vaccinations [18, 33]. 

#### Reminders

We have higher confidence in reminders to physicians to vaccinate [13], and medium confidence in the use of computerised reminders to providers to vaccinate (four reviews) [16, 18, 33, 35]. These are reminders which are sent or flagged to health care professionals to alert them to the need to vaccinate, rather than reminders to patients to attend for vaccination.

#### Personalised electronic health records

These were not evaluated as a standalone intervention, but we have medium confidence that their use, together with their electronic messaging features, to educate, send reminders and schedule appointments may increase vaccination relative to control groups with only record access or with no access, including where postal reminders were used [15]. 

#### Other low intensity approaches

There is low to moderate certainty evidence from the Cochrane review in influenza vaccination for older people that the following may be effective: payments to physicians; reminding physicians to vaccinate all patients; posters in clinics presenting vaccination rates and encouraging competition between doctors; chart reviews and benchmarking to rates achieved by the top 10% of physicians [13]. 

## Discussion

### Summary of the evidence

We identified 23 systematic reviews which included RCTs of interventions to increase vaccination in vulnerable groups. Of these 18 reviews were published after the 2015 overview of reviews identified in our scoping work [4]. In this overview we have summarised randomised evidence for high, medium and low intensity examples of interventions to increase demand for vaccination; interventions to increase access to vaccination; and provider-focused interventions.

The best represented interventions targeted vaccination demand. We had higher confidence in the effectiveness of high, medium and low intensity communication interventions: home visits, telephone calls and text messages respectively. We had higher confidence in home visits for vaccination but medium confidence in evidence for other interventions for increasing access, including additional clinics. We did not identify patient-focused medium or low intensity interventions to increase access to vaccination. However there were provider-focused interventions which would likely have increased access, such as changing systems to allow vaccination on routine or unrelated visits. For provider-focused interventions we had higher confidence in facilitator visits to practices (high intensity incorporating lower intensity components) and centralised reminder/recall systems (low intensity), and medium confidence in case management (moderate intensity). Where interventions did not show evidence of an effect we typically had lower confidence in the evidence.

## Strengths and limitations: review process

We searched multiple databases using a strategy designed by an information specialist and updated the search in November 2022 to capture rapidly developing literature in the context of the COVID-19 pandemic. We have surveyed the literature published since then to further contextualise the relevance of the review. We limited our overview to reviews published in English, but the eight identified reviews in other languages would have been excluded for other reasons. We undertook this overview rapidly to inform work to increase vaccination uptake among vulnerable groups in Greater Manchester. We therefore did not screen all records in duplicate, but we used duplicate screening for a sample of records and for all records where there was uncertainty; two reviewers evaluated most full text records because decisions were nuanced; and we checked samples of data extraction. Two researchers agreed risk of bias and confidence assessments and undertook ROBIS assessments independently. We prespecified our synthesis approach to categorising interventions and strength of evidence and informed this using GROOVE mapping of overlap between reviews.

While our approach to categorising evidence was based on those of other reviews [12, 13], it was necessarily subjective to some degree. We partially mitigated this by having two reviewers involved in the categorisation process and discussing disagreements within the review team. However, many interventions will contain elements of more than one category even when they are not multicomponent. In particular interventions which involve providers sending recalls or reminders to patients can be conceived of as both intended to increase demand for vaccination (targeting pull factors) and as provider focused. Categorisation of these was based on the intervention description and whether the focus was on the communication with the patient or the providers’ systems to enable these. We acknowledge that this distinction is to some degree arbitrary. Finally we recognise that our categorisation of intensity is based, as in the review that used it previously [13], on the resource implications of the intervention for providers. While it is less resource intensive to send text messages than to make phone calls to patients, patients may experience (for example) a series of repeated and tailored text messages as a more intensive intervention than a single generic phone call. The patient experience of intervention intensity is outside the scope of this work but would be worth exploring further.

Our dependence the conduct and reporting of included reviews limited us in multiple respects. In some reviews information was extremely limited and we did not have capacity to directly check relevance of primary studies for study design, intervention, population and outcomes if this was not apparent. In one review, we were unsure how many relevant RCTs were included [30]. This may have led to exclusion of some relevant evidence. Overlap between reviews means evidence missed in one review may be identified elsewhere. We also did not have capacity to check risk of bias assessments or conduct them where they were absent; lack of assessments reduced our confidence in evidence.

## Strengths and limitations: scope of review

We limited this overview to randomised evidence and so did not include specific interventions only evaluated by non-randomised studies, and some included interventions are only represented by small numbers of RCTs or RCTs with small numbers of participants. We are conscious that some important interventions, such as the class of societal interventions identified in Thomas (2018), have thereby been excluded entirely [13]. This decision also meant that we included only very limited evidence for people from LGBTQ + communities; only one review included relevant RCTs [27]. We accept that limitation to RCTs is also likely to have excluded interventions which are evaluated in other ways, which can be appropriate research designs in the context of the work, often in partnership with a community, which is being undertaken. We have undertaken such work ourselves in Greater Manchester [37], informed in part by this review, and would suggest that reading our review in the context of reports of this work – which may be found in the grey as much as the published literature – would be appropriate.

We have not identified RCT evidence not included in a systematic review indexed by 2022; this is a necessary constraint in a review of reviews. However, we updated the Medline search in April 2024 to assess the impact of this cutoff on the review. A large number of reviews published or indexed after December 2022 evaluated vaccination uptake and barriers and facilitators to this in both general populations and minority or otherwise vulnerable groups in the context of COVID-19 vaccination programmes. However, only three reviews would have been eligible for inclusion in out review [38–40]. 

The most substantive evidence was supplied by a review of behaviour change techniques in minority ethnic populations. This included ten RCTs and reported that across all study designs and multiple target vaccines the most commonly used intervention functions were education, persuasion and enablement. Effective interventions were multicomponent and tailored to the target population, while awareness raising and community organisation involvement were also associated with positive effects [39]. We suggest that this review be read in conjunction with our overview. Two other reviews each included a small number of relevant RCTs. One contained a single relevant RCT relating to willingness to receive COVID-19 vaccination among black and minority ethnic people in the UK and explored the effectiveness of exposure to different forms of written information [40]. Another contained two relevant RCTs targeting influenza vaccination in older adults with or without additional markers of vulnerability or marginalisation [38]. While we would include these reviews in an update of this overview we do not consider that they are likely to substantively change our findings.

We also identified very limited evidence relating to financial incentives for vaccination, this was always part of a wider intervention, and we did not deal with it separately. Free vaccination was evaluated but is not included because our overview was undertaken to inform COVID-19 vaccination work in the UK where universal free vaccination was available.

We excluded several recent scoping reviews, which may be more up to date than systematic reviews. The most substantive is a Cochrane scoping review of interventions for COVID-19 vaccine hesitancy [41]. This was not limited to minoritised or vulnerable groups although some included studies focused on them. Of the 61 completed studies identified, none were systematic reviews and 45 were RCTs; these focused on online communication interventions posing hypothetical decision-making scenarios. Thirty-five ongoing studies (29 RCTs) mainly evaluated education or communication interventions. An update or subsequent systematic review may identify completed trials relevant to our overview.

### Applicability

Identified evidence relates to specific groups and its transferability to other marginalised or vulnerable groups is not evidenced. A substantial amount of the evidence comes from people who are vulnerable due to older age. We did not identify any evidence relating to minoritised faith groups. Some evidence related to people from minority ethnic groups, who may also be members of minority faith groups, but interventions were not targeted on this basis and most evidence related to African or Hispanic Americans who are often members of majority faiths. We therefore did not find evidence for interventions such as women-only vaccination sessions targeted at Muslim or Orthodox Jewish communities.

## Conclusions and further research

Considerable evidence supports the probable effectiveness of communication in person, by phone or in writing to increase vaccination; this includes evidence from a Cochrane review with an overall GRADE assessment of moderate certainty. Both high and low intensity interventions targeting providers showed increases in vaccination compared to standard care. However, our overview highlighted the comparatively very limited evidence assessing key strategies to increase access, such as additional clinics or targeted services for increasing access. Only the very high intensity intervention of home visits had higher confidence evidence showing effectiveness. None of the evidence related to COVID-19 vaccination where inequalities of outcome are exacerbated.

There was no evidence for interventions for religious minority communities; this may intersect with gaps in evidence for additional services. Systematic reviews looking specifically at interventions targeting these communities may be needed. We identified very limited evidence for online messaging, video messaging or mass media messaging. Following the COVID-19 pandemic these approaches are not yet well-represented in systematic reviews and a systematic review of primary evidence for these types of communication may also be warranted.

We identified many reviews of barriers and facilitators for vaccination, often relevant to vulnerable or minoritised groups. We also identified several reviews of vaccination programmes. Both sets of reviews would be of interest to those designing interventions to increase vaccination uptake. We did not identify overviews of reviews in either area and there may be merit in undertaking these. We identified multiple, often overlapping reviews in a rapidly growing research field. It may therefore be useful to establish a living systematic review of trials, and to encourage trialists to collaborate actively with the reviewers.

### Electronic supplementary material

Below is the link to the electronic supplementary material.

**Supplementary Material 1 Appendix 1**: Full search strategies

**Supplementary Material 2 Appendix 2**: Documentation of evidence for interventions

## Data Availability

All data were previously published; full search strategies and data coding used to support the synthesis are provided in supplementary material or in the main text. Additional data are available on request.

## References

[1] Butler R, McDonald NE (2015). SAGE Working Group on Vaccine Hesitancy. Diagnosing the determinants of vaccine hesitancy in specific subgroups: the Guide to Tailoring immunization programmes (TIP). Vaccine.

[2] Watkinson RE (2022). Ethnic inequalities in COVID-19 vaccine uptake and comparison to seasonal influenza vaccine uptake in Greater Manchester, UK: a cohort study. PLoS Med.

[3] *Disparities in the risk and outcomes of COVID-19*. 2020, Public Health England: https://assets.publishing.service.gov.uk/government/uploads/system/uploads/attachment_data/file/908434/Disparities_in_the_risk_and_outcomes_of_COVID_August_2020_update.pdf.

[4] Dubé E, Gagnon D, McDonald NE (2015). Strategies intended to address vaccine hesitancy: review of published reviews. Vaccine.

[5] Norman G, Kletter M, Dumville J. *CRD42021293355. Rapid review of reviews of interventions for vaccine hesitancy in underserved, minority or vulnerable groups*. 2021.

[6] Gates M (2022). Reporting guideline for overviews of reviews of healthcare interventions: development of the PRIOR statement. BMJ.

[7] The EndNote Team (2013). EndNote.

[8] Ouzzani M (2016). Rayyan—a web and mobile app for systematic reviews. Syst Reviews.

[9] Guyatt G, Oxman A, Akl E (2011). GRADE guidelines: 1. Introduction-GRADE evidence profiles and Summary of findings tables. J Clin Epidemiol.

[10] Whiting P, Savović J, Higgins JP (2016). ROBIS: a new tool to assess risk of bias in systematic reviews was developed. J Clin Epidemiol.

[11] Campbell M, McKenzie JE, Sowden A (2020). Synthesis without meta-analysis (SWiM) in systematic reviews: reporting guideline. BMJ.

[12] *Strategies to improve vaccination uptake in Australia, a systematic review of types and effectiveness… corrected] [published erratum appears in AUST NZ J PUBLIC HEALTH 2012; 36(5):490]* Australian & New Zealand Journal of Public Health, 2012. 36(4): pp. 369–377.

[13] Thomas RE, Lorenzetti DL (2018). Interventions to increase influenza vaccination rates of those 60 years and older in the community. Cochrane Database Syst Reviews.

[14] Pérez-Bracchiglione J (2022). Graphical representation of overlap for OVErviews: GROOVE tool. Res Synthesis Methods.

[15] Balzarini F (2020). Does the use of personal electronic health records increase vaccine uptake? A systematic review. Vaccine.

[16] Brandt HM (2021). A narrative review of HPV vaccination interventions in rural U.S. communities. Prev Med.

[17] Callahan AG (2021). Racial disparities in influenza immunization during pregnancy in the United States: a narrative review of the evidence for disparities and potential interventions. Vaccine.

[18] Crocker-Buque T, Edelstein M, Mounier-Jack S (2017). Interventions to reduce inequalities in vaccine uptake in children and adolescents aged < 19 years: a systematic review. J Epidemiol Community Health.

[19] Crocker-Buque T (2017). Immunization, urbanization and slums - a systematic review of factors and interventions. BMC Public Health.

[20] Glenton C (2011). Can lay health workers increase the uptake of childhood immunisation? Systematic review and typology. Tropical Med Int Health.

[21] Gopalani SV (2022). Barriers and Factors Associated with HPV Vaccination among American indians and Alaska Natives: a systematic review. J Community Health.

[22] Isenor JE (2016). Impact of pharmacists as immunizers on vaccination rates: a systematic review and meta-analysis. Vaccine.

[23] Kaufman J et al. Face-to‐face interventions for informing or educating parents about early childhood vaccination. Cochrane Database Syst Reviews, 2018(5). 10.1002/14651858.CD010038.pub3 PMC6494431 29736980

[24] Kendrick D (2000). The effect of home visiting programmes on uptake of childhood immunization: a systematic review and meta-analysis. J Public Health Med.

[25] Lambert JF (2021). Reducing burden from respiratory infections in refugees and immigrants: a systematic review of interventions in OECD, EU, EEA and EU-applicant countries. BMC Infect Dis.

[26] Lewin S, et al. Lay health workers in primary and community health care for maternal and child health and the management of infectious diseases. Cochrane Database of Systematic Reviews; 2010. 3. 10.1002/14651858.CD004015.pub3 PMC6485809 20238326

[27] Lott BE (2020). Interventions to increase uptake of human papillomavirus (HPV) vaccination in minority populations: a systematic review. Prev Med Rep.

[28] Machado AA (2021). Effective interventions to increase routine childhood immunization coverage in low socioeconomic status communities in developed countries: a systematic review and critical appraisal of peer-reviewed literature. Vaccine.

[29] Mogaka EN (2019). Effectiveness of an educational intervention to increase human papillomavirus knowledge in high-risk populations: a systematic review. Univ Tor Med J.

[30] Mohammed H (2021). A rapid global review of strategies to improve influenza vaccination uptake in Australia. Hum Vaccines Immunotherapeutics.

[31] Murray E (2021). Impact of pharmacy intervention on influenza vaccination acceptance: a systematic literature review and meta-analysis. Int J Clin Pharm.

[32] Nelson KN et al. Assessing strategies for increasing urban routine immunization coverage of childhood vaccines in low and middle-income countries: a systematic review of peer-reviewed literature. Vaccine, 2016. 34(5495 – 503). 10.1016/j.vaccine.2016.09.038 PMC5309783 27692772

[33] Odone A, et al. Effectiveness of interventions that apply new media to improve vaccine uptake and vaccine coverage. Volume 11. Human vaccines & Immunotherapeutics; 2015. pp. 72–82. 1. 10.4161/hv.34313 PMC4514191 25483518

[34] Rani U (2022). Public Education Interventions and Uptake of Human Papillomavirus Vaccine: a systematic review. J Public Health Manage Pract.

[35] Sarnoff R, Rundall T (1998). Meta-analysis of effectiveness of interventions to increase influenza immunization rates among high-risk population groups. Med Care Res Rev.

[36] Vedio A (2017). Improving access to health care for chronic hepatitis B among migrant Chinese populations: a systematic mixed methods review of barriers and enablers. J Viral Hepatitis.

[37] Bradley F et al. *Optimising Targeted Vaccination Activity in Greater Manchester*. 2023, NIHR Applied Research Collaboration Greater Manchester: https://arc-gm.nihr.ac.uk/media/Resources/ARC/Evaluation/NIPP%20-%20Vaccine%20Optimitsation/NIPP%20Report%20-%20FINAL.pdf.

[38] Gobbo ELS (2023). Do peer-based education interventions effectively improve vaccination acceptance? A systematic review. BMC Public Health.

[39] Ekezie W et al. *A Systematic Review of Behaviour Change Techniques within Interventions to Increase Vaccine Uptake among Ethnic Minority Populations* Vaccines (Basel), 2023. 11(7). 10.3390/vaccines11071259 PMC10386142 37515074

[40] Hussain B (2022). Overcoming COVID-19 vaccine hesitancy among ethnic minorities: a systematic review of UK studies. Vaccine.

[41] Andreas M et al. Interventions to increase COVID-19 vaccine uptake: a scoping review. Cochrane Database Syst Reviews, 2022(8). 10.1002/14651858.CD015270 PMC9347311 35920693

