# Supplementary material for: Interventions to increase vaccination in vulnerable groups: rapid overview of reviews
Source: BMC Public Health. 2024 Jun 3;24:1479. doi: 10.1186/s12889-024-18713-5 (PMC11145854; doi:10.1186/s12889-024-18713-5)
Supplement: Supplementary file 1 — Supplementary Material 1 Appendix 1: Full search strategies [file 12889_2024_18713_MOESM1_ESM.docx]

# Appendix 1: Search strategies

**Cochrane Database of Systematic Reviews (CDSR) in the Cochrane Library**

#1 ((vaccin* or immunis* or immuniz*) near/5 (anxiety or attitude* or awareness or behavio?r or belief* or criticis* or doubt* or distrust or dropout* or exemption* or fear* or hesitanc* or trust or mistrust or perception* or refus* or rejection or rumo?r* or intent* or controvers* or misconception* or misinformation or opposition or delay or dilemma* or objector*)):ti,ab,kw

#2 ((vaccin* or immunis* or immuniz*) near/3 (uptake or barrier* or choice* or mandatory or compulsory or concern* or accepta* or knowledge or parent* con*)):ti,ab,kw

#3 ((vaccin* or immunis* or immuniz*) near/5 confidence):ti,ab,kw

#4 ((vaccin* or immunis* or immuniz*) near/5 decision making):ti,ab,kw

#5 ((vaccin* or immunis* or immuniz*) and (anti-vaccin* or antivaccin*)):ti,ab,kw

#6 {Or #1-#5}

#7 MeSH descriptor: [Vaccination] explode all trees

#8 MeSH descriptor: [Vaccines] explode all trees

#9 MeSH descriptor: [Immunization] explode all trees

#10 MeSH descriptor: [Immunization Programs] explode all trees

#11 {Or #7-#10}

#12 MeSH descriptor: [Public Opinion] explode all trees

#13 MeSH descriptor: [Attitude to Health] explode all trees

#14 MeSH descriptor: [Attitude] this term only

#15 MeSH descriptor: [Patient Acceptance of Health Care] explode all trees

#16 MeSH descriptor: [Treatment Refusal] explode all trees

#17 MeSH descriptor: [Parental Consent] explode all trees

#18 MeSH descriptor: [Decision Making] explode all trees

#19 MeSH descriptor: [Prejudice] this term only

#20 MeSH descriptor: [Internet] this term only

#21 {or #12-#20}

#22 #11 and #21

#23 #6 or #22

#24 MeSH descriptor: [Vulnerable Populations] explode all trees

#25 MeSH descriptor: [Minority Groups] explode all trees

#26 MeSH descriptor: [Medically Underserved Area] explode all trees

#27 MeSH descriptor: [Disabled Persons] explode all trees

#28 ((underserve* or disadvantage* or minorit* or marginali*) near/6 (group* or population*)):ti,ab,kw

#29 (low pay or low paid or low income or unemploy* or depriv* or financial hardship or benefit recipient* or social position* or social class* or socioeconomic or social status or poverty or impoverish*):ti,ab,kw

#30 MeSH descriptor: [Poverty] explode all trees

#31 MeSH descriptor: [Health Status] explode all trees

#32 MeSH descriptor: [Population Groups] explode all trees

#33 MeSH descriptor: [Minority Health] explode all trees

#34 MeSH descriptor: [Multilingualism] explode all trees

#35 MeSH descriptor: [Refugees] explode all trees

#36 MeSH descriptor: [Cultural Characteristics] explode all trees

#37 MeSH descriptor: [Islam] explode all trees

#38 MeSH descriptor: [Hinduism] explode all trees

#39 MeSH descriptor: [Judaism] explode all trees

#40 MeSH descriptor: [Christianity] explode all trees

#41 MeSH descriptor: [Buddhism] explode all trees

#42 (black or hispanic* or ((afr* or asian or latin* or indian) near/1 american*)):ti,ab,kw

#43 (black or black british):ti,ab,kw

#44 ("black asian and minority ethnic" or BAME):ti,ab,kw

#45 (black near/3 (afr* or caribbean or british)):ti,ab,kw

#46 (south near/3 asian*):ti,ab,kw

#47 (("people of color") or ("people of colour")):ti,ab,kw

#48 (rural or refugee* or immigrant* or ethnic minorit* or ethnic* or racial or indigenous or first nation):ti,ab,kw

#49 (islam* or hindu* or sikh* or buddhism* or christian* or judaism or muslim):ti,ab,kw

#50 ((travel* or roma* or gyps*) near/3 (communit* or famil*)):ti,ab,kw

#51 ((mental* or intellectual*) near/2 (impair* or disab* or defici* or handicap*)):ti,ab,kw

#52 ((learning* or cognit*) near/2 (impair* or disab* or defici* or difficulty or difficulties or disorder* or handicap*)):ti,ab,kw

#53 ((low* or handicap* or impair* or partial* or disab* or disorder* or loss* or limit*) near/3 (vision or visual* or sight*)):ti,ab,kw

#54 (hearing near/2 (loss* or impair* or partial* or deficit* or deteriorat* or degenerat* or diminish* or difficult* or disabilit* or hard or one side* or unilateral)):ti,ab,kw

#55 deaf:ti,ab,kw

#56 ((physical* or mobility) near/2 (impair* or disab* or defici* or difficulty or difficulties or disorder* or handicap*)):ti,ab,kw

#57 MeSH descriptor: [Aged] explode all trees

#58 ("50 years or older" or "55 years or older" or "60 years or older" or "65 years or older" or "70 years or older" or "75 years or older" or "80 years or older"):ti,ab,kw

#59 ("older than 50" or "older than 55" or "older than 60" or "older than 65" or "older than 70" or "older than 75" or "older than 80"):ti,ab,kw

#60 {OR #24-#59}

#61 #23 and #60 in Cochrane Reviews

**Ovid MEDLINE**

1 ((vaccin* or immunis* or immuniz*) adj5 (anxiety or attitude* or awareness or behavio?r or belief* or criticis* or doubt* or distrust or dropout* or exemption* or fear* or hesitanc* or low* or trust or mistrust or perception* or refus*5 or rejection or rumo?r* or intent*5 or controvers* or misconception* or misinformation or opposition or delay or dilemma* or objector*)).ti,ab.

2 ((vaccin* or immunis* or immuniz*) adj3 (uptake or barrier* or choice* or mandatory or compulsory or concern* or accepta* or knowledge or parent* con*)).ti,ab.

3 (((vaccin* or immunis* or immuniz*) adj5 confidence) not confidence interval).ti,ab.

4 ((vaccin* or immunis* or immuniz*) adj5 decision making).ti,ab.

5 ((vaccin* or immunis* or immuniz*) and (anti-vaccin* or antivaccin*)).ti,ab.

6 1 or 2 or 3 or 4 or 5

7 exp vaccination/

8 exp Vaccines/

9 exp Immunization/

10 exp Immunization Programs/

11 or/7-10

12 exp Public Opinion/

13 exp Attitude to Health/

14 Attitude/

15 exp "Patient Acceptance of Health Care"/

16 exp Treatment Refusal/

17 exp Parental Consent/

18 exp Decision Making/

19 Prejudice/

20 Internet/

21 or/12-20

22 11 and 21

23 6 or 22

24 exp Vulnerable Populations/

25 exp Minority Groups/

26 exp Medically Underserved Area/

27 exp Disabled Persons/

28 ((underserve* or disadvantage* or minorit* or marginali*) adj6 (group* or population*)).ti,ab.

29 (low pay or low paid or low income or unemploy* or depriv* or financial hardship or benefit recipient* or social position* or social class* or socioeconomic or social status or poverty or impoverish*).ti,ab.

30 exp Poverty/

31 exp Health Status/

32 exp Population Groups/

33 exp Minority Health/

34 exp multilingualism/

35 exp Refugees/

36 exp Cultural Characteristics/

37 exp Islam/

38 exp Hinduism/

39 exp Judaism/

40 exp Christianity/

41 exp Buddhism/

42 (black or hispanic* or ((afr* or asian or latin* or indian) adj1 american*)).ti,ab. 241095

43 (black or black british).ti,ab.

44 ("black asian and minority ethnic" or BAME).ti,ab.

45 (black adj3 (afr* or caribbean or british)).ti,ab.

46 (south adj3 asian*).ti,ab.

47 people of colo?r.ti,ab.

48 (rural or refugee* or immigrant* or ethnic minorit* or ethnic* or racial or indigenous or first nation).ti,ab.

49 (islam* or hindu* or sikh* or buddhism* or christian* or judaism or muslim).ti,ab.

50 ((travel* or roma* or gyps*) adj3 (communit* or famil*)).ti,ab.

51 ((mental* or intellectual*) adj2 (impair* or disab* or defici* or handicap*)).ti,ab.

52 ((learning* or cognit*) adj2 (impair* or disab* or defici* or difficulty or difficulties or disorder* or handicap*)).ti,ab.

53 ((low* or handicap* or impair* or partial* or disab* or disorder* or loss* or limit*) adj3 (vision or visual* or sight*)).ti,ab.

54 (hearing adj2 (loss* or impair* or partial* or deficit* or deteriorat* or degenerat* or diminish* or difficult* or disabilit* or hard or one side* or unilateral)).ti,ab.

55 deaf.ti,ab.

56 ((physical* or mobility) adj2 (impair* or disab* or defici* or difficulty or difficulties or disorder* or handicap*)).ti,ab.

57 exp Aged/

58 ("50 years or older" or "55 years or older" or "60 years or older" or "65 years or older" or "70 years or older" or "75 years or older" or "80 years or older").ti,ab.

59 ("older than 50" or "older than 55" or "older than 60" or "older than 65" or "older than 70" or "older than 75" or "older than 80").ti,ab.

60 or/24-59

61 23 and 60

62 exp Meta-Analysis as Topic/

63 meta analy$.tw.

64 metaanaly$.tw.

65 meta-analysis/

66 (systematic adj (review$1 or overview$1)).tw.

67 exp Review Literature as Topic/

68 62 or 63 or 64 or 65 or 66 or 67

69 cochrane.ab.

70 embase.ab.

71 (psychlit or psyclit).ab.

72 (psychinfo or psycinfo).ab.

73 (cinahl or cinhal).ab.

74 science citation index.ab.

75 bids.ab.

76 cancerlit.ab.

77 69 or 70 or 71 or 72 or 73 or 74 or 75 or 76

78 reference list$.ab.

79 bibliograph$.ab.

80 hand-search$.ab.

81 relevant journals.ab.

82 manual search$.ab.

83 78 or 79 or 80 or 81 or 82

84 selection criteria.ab.

85 data extraction.ab.

86 84 or 85

87 Review/

88 86 and 87

89 comment/

90 letter/

91 editorial/

92 animal/

93 human/

94 92 not (92 and 93)

95 89 or 90 or 91 or 94

96 68 or 77 or 83 or 88

97 96 not 95

98 61 and 97

**Ovid Embase**

1 ((vaccin* or immunis* or immuniz*) adj5 (anxiety or attitude* or awareness or behavio?r or belief* or criticis* or doubt* or distrust or dropout* or exemption* or fear* or hesitanc* or low* or trust or mistrust or perception* or refus*5 or rejection or rumo?r* or intent*5 or controvers* or misconception* or misinformation or opposition or delay or dilemma* or objector*)).ti,ab.

2 ((vaccin* or immunis* or immuniz*) adj3 (uptake or barrier* or choice* or mandatory or compulsory or concern* or accepta* or knowledge or parent* con*)).ti,ab.

3 (((vaccin* or immunis* or immuniz*) adj5 confidence) not confidence interval).ti,ab.

4 ((vaccin* or immunis* or immuniz*) adj5 decision making).ti,ab.

5 ((vaccin* or immunis* or immuniz*) and (anti-vaccin* or antivaccin*)).ti,ab.

6 1 or 2 or 3 or 4 or 5

7 exp vaccination/

8 exp Vaccines/

9 exp Immunization/

10 or/7-9

11 exp Public Opinion/

12 exp Attitude to Health/

13 Attitude/

14 exp patient attitude/

15 exp Treatment Refusal/

16 exp Parental Consent/

17 exp Decision Making/

18 Prejudice/

19 Internet/

20 or/11-19

21 10 and 20

22 6 or 21

23 exp Vulnerable Populations/

24 exp Minority Groups/

25 exp Medically Underserved Area/

26 exp disabled person/

27 ((underserve* or disadvantage* or minorit* or marginali*) adj6 (group* or population*)).ti,ab.

28 (low pay or low paid or low income or unemploy* or depriv* or financial hardship or benefit recipient* or social position* or social class* or socioeconomic or social status or poverty or impoverish*).ti,ab.

29 exp Poverty/

30 exp Health Status/

31 population group/

32 exp Minority Health/

33 exp multilingualism/

34 exp refugee/

35 exp cultural factor/

36 exp Islam/

37 exp Hinduism/

38 exp Judaism/

39 exp Christianity/

40 exp Buddhism/

41 (black or hispanic* or ((afr* or asian or latin* or indian) adj1 american*)).ti,ab.

42 (black or black british).ti,ab.

43 ("black asian and minority ethnic" or BAME).ti,ab.

44 (black adj3 (afr* or caribbean or british)).ti,ab.

45 (south adj3 asian*).ti,ab.

46 people of colo?r.ti,ab.

47 (rural or refugee* or immigrant* or ethnic minorit* or ethnic* or racial or indigenous or first nation).ti,ab.

48 (islam* or hindu* or sikh* or buddhism* or christian* or judaism or muslim).ti,ab.

49 ((travel* or roma* or gyps*) adj3 (communit* or famil*)).ti,ab.

50 ((mental* or intellectual*) adj2 (impair* or disab* or defici* or handicap*)).ti,ab.

51 ((learning* or cognit*) adj2 (impair* or disab* or defici* or difficulty or difficulties or disorder* or handicap*)).ti,ab.

52 ((low* or handicap* or impair* or partial* or disab* or disorder* or loss* or limit*) adj3 (vision or visual* or sight*)).ti,ab.

53 (hearing adj2 (loss* or impair* or partial* or deficit* or deteriorat* or degenerat* or diminish* or difficult* or disabilit* or hard or one side* or unilateral)).ti,ab.

54 deaf.ti,ab.

55 ((physical* or mobility) adj2 (impair* or disab* or defici* or difficulty or difficulties or disorder* or handicap*)).ti,ab.

56 exp Aged/

57 ("50 years or older" or "55 years or older" or "60 years or older" or "65 years or older" or "70 years or older" or "75 years or older" or "80 years or older").ti,ab.

58 ("older than 50" or "older than 55" or "older than 60" or "older than 65" or "older than 70" or "older than 75" or "older than 80").ti,ab.

59 or/23-58

60 22 and 59

61 exp Meta Analysis/

62 ((meta adj analy$) or metaanalys$).tw.

63 (systematic adj (review$1 or overview$1)).tw.

64 61 or 62 or 63

65 cancerlit.ab.

66 cochrane.ab.

67 embase.ab.

68 (psychlit or psyclit).ab.

69 (psychinfo or psycinfo).ab.

70 (cinahl or cinhal).ab.

71 science citation index.ab.

72 bids.ab.

73 65 or 66 or 67 or 68 or 69 or 70 or 71 or 72

74 reference lists.ab.

75 bibliograph$.ab.

76 hand-search$.ab.

77 manual search$.ab.

78 relevant journals.ab.

79 74 or 75 or 76 or 77 or 78

80 data extraction.ab.

81 selection criteria.ab.

82 80 or 81

83 review.pt.

84 82 and 83

85 letter.pt.

86 editorial.pt.

87 animal/

88 human/

89 87 not (87 and 88)

90 85 or 86 or 89

91 64 or 73 or 79 or 84

92 91 not 90

93 60 and 92

**EBSCO CINAHL Plus**

S74 S61 AND S73

S73 S67 NOT S72

S72 S68 OR S69 OR S70 OR S71

S71 (MH "Animals")

S70 PT editorial

S69 PT letter

S68 PT commentary

S67 S62 OR S63 OR S64 OR S65 OR S66

S66 TI ( systematic review or systematic overview ) OR AB ( systematic review or systematic overview

S65 (MH "Literature Review+")

S64 TI Metaanalys* OR AB Metaanalys*

S63 TI Meta analys* OR AB Meta analys*

S62 (MH "Meta Analysis")

S61 S22 AND S60

S60 S23 OR S24 OR S25 OR S26 OR S27 OR S28 OR S29 OR S30 OR S31 OR S32 OR S33 OR S34 OR S35 OR S36 OR S37 OR S38 OR S39 OR S40 OR S41 OR S42 OR S43 OR S44 OR S45 OR S46 OR S47 OR S48 OR S49 OR S50 OR S51 OR S52 OR S53 OR S54 OR S55 OR S56 OR S57 OR S58 OR S59

S59 TI ( ("older than 50" or "older than 55" or "older than 60" or "older than 65" or "older than 70" or "older than 75" or "older than 80") ) OR AB ( ("older than 50" or "older than 55" or "older than 60" or "older than 65" or "older than 70" or "older than 75" or "older than 80") )

S58 TI ( ("50 years or older" or "55 years or older" or "60 years or older" or "65 years or older" or "70 years or older" or "75 years or older" or "80 years or older") ) OR AB ( ("50 years or older" or "55 years or older" or "60 years or older" or "65 years or older" or "70 years or older" or "75 years or older" or "80 years or older") )

S57 (MH "Aged+")

S56 TI ( ((physical* or mobility) N2 (impair* or disab* or defici* or difficulty or difficulties or disorder* or handicap*)) ) OR AB ( ((physical* or mobility) N2 (impair* or disab* or defici* or difficulty or difficulties or disorder* or handicap*)) )

S55 TI deaf OR AB deaf

S54 TI ( (hearing N2 (loss* or impair* or partial* or deficit* or deteriorat* or degenerat* or diminish* or difficult* or disabilit* or hard or one side* or unilateral)) ) OR AB ( (hearing N2 (loss* or impair* or partial* or deficit* or deteriorat* or degenerat* or diminish* or difficult* or disabilit* or hard or one side* or unilateral)) )

S53 TI ( ((low* or handicap* or impair* or partial* or disab* or disorder* or loss* or limit*) N3 (vision or visual* or sight*)) ) OR AB ( ((low* or handicap* or impair* or partial* or disab* or disorder* or loss* or limit*) N3 (vision or visual* or sight*)) )

S52 TI ( ((learning* or cognit*) N2 (impair* or disab* or defici* or difficulty or difficulties or disorder* or handicap*)) ) OR AB ( ((learning* or cognit*) N2 (impair* or disab* or defici* or difficulty or difficulties or disorder* or handicap*)) )

S51 TI ( ((mental* or intellectual*) N2 (impair* or disab* or defici* or handicap*)) ) OR AB ( ((mental* or intellectual*) N2 (impair* or disab* or defici* or handicap*)) )

S50 TI ( ((travel* or roma* or gyps*) N3 (communit* or famil*)) ) OR AB ( (islam* or hindu* or ((travel* or roma* or gyps*) N3 (communit* or famil*)) )

S49 TI ( (islam* or hindu* or sikh* or buddhism* or christian* or judaism or muslim) ) OR AB ( (islam* or hindu* or sikh* or buddhism* or christian* or judaism or muslim) )

S48 TI ( (rural or refugee* or immigrant* or ethnic minorit* or ethnic* or racial or indigenous or first nation) ) OR AB ( (rural or refugee* or immigrant* or ethnic minorit* or ethnic* or racial or indigenous or first nation) )

S47 TI “people of colo#r” OR AB “people of colo#r”

S46 TI (south N3 asian*) OR AB (south N3 asian*)

S45 TI ( (black N3 (afr* or caribbean or british)) ) OR AB ( (black N3 (afr* or caribbean or british)) )

S44 TI ( ("black asian and minority ethnic" or BAME) ) OR AB ( ("black asian and minority ethnic" or BAME) )

S43 TI ( (black or black british) ) OR AB ( (black or black british) )

S42 TI ( (black or hispanic* or ((afr* or asian or latin* or indian) N1 american*)) ) OR AB ( (black or hispanic* or ((afr* or asian or latin* or indian) N1 american*)) )

S41 (MH "Buddhism")

S40 (MH "Christianity+")

S39 (MH "Judaism")

S38 (MH "Hinduism")

S37 (MH "Islam")

S36 (MH "Cultural Values")

S35 (MH "Refugees+")

S34 (MH "Multilingualism")

S33 (MH "Ethnic Groups+")

S32 (MH "Health Status+")

S31 (MH "Poverty+")

S30 TI ( (low pay or low paid or low income or unemploy* or depriv* or financial hardship or benefit recipient* or social position* or social class* or socioeconomic or social status or poverty or impoverish*) ) OR AB ( (low pay or low paid or low income or unemploy* or depriv* or financial hardship or benefit recipient* or social position* or social class* or socioeconomic or social status or poverty or impoverish*) )

S29 TI ( ((underserve* or disadvantage* or minorit* or marginali*) N6 (group* or population*)) ) OR AB ( ((underserve* or disadvantage* or minorit* or marginali*) N6 (group* or population*)) )

S28 (MH "Disabled+")

S27 (MH "Medically Underserved Area")

S26 (MH "Medically Underserved")

S25 (MH "Minority Groups")

S24 (MH "Vulnerability")

S23 (MH "Special Populations")

S22 S6 OR S21

S21 S10 AND S20

S20 S11 OR S12 OR S13 OR S14 OR S15 OR S16 OR S17 OR S18 OR S19

S19 (MH "Internet")

S18 (MH "Prejudice")

S17 (MH "Decision Making+")

S16 (MH "Consent+")

S15 (MH "Treatment Refusal")

S14 (MH "Patient Attitudes")

S13 (MH "Attitude")

S12 (MH "Attitude to Health+")

S11 (MH "Public Opinion")

S10 S7 OR S8 OR S9

S9 (MH "Immunization Programs")

S8 (MH "Vaccines+")

S7 (MH "Immunization+")

S6 S1 OR S2 OR S4 OR S5

S5 TI ( ((vaccin* or immunis* or immuniz*) and (anti-vaccin* or antivaccin*)) ) OR AB ( ((vaccin* or immunis* or immuniz*) and (anti-vaccin* or antivaccin*)) )

S4 TI ( ((vaccin* or immunis* or immuniz*) N5 decision making) ) OR AB ( ((vaccin* or immunis* or immuniz*) N5 decision making) )

S3 TI ( (((vaccin* or immunis* or immuniz*) N5 confidence) not confidence interval) ) OR AB ( (((vaccin* or immunis* or immuniz*) N5 confidence) not confidence interval) )

S2 TI ( ((vaccin* or immunis* or immuniz*) N3 (uptake or barrier* or choice* or mandatory or compulsory or concern* or accepta* or knowledge or parent* con*)) ) OR AB ( ((vaccin* or immunis* or immuniz*) N3 (uptake or barrier* or choice* or mandatory or compulsory or concern* or accepta* or knowledge or parent* con*)) )

S1 TI ( ((vaccin* or immunis* or immuniz*) N5 (anxiety or attitude* or awareness or behavio#r or belief* or criticis* or doubt* or distrust or dropout* or exemption* or fear* or hesitanc* or low* or trust or mistrust or perception* or refus* or rejection or rumo#r* or intent* or controvers* or misconception* or misinformation or opposition or delay or dilemma* or objector*)) ) OR AB ( ((vaccin* or immunis* or immuniz*) N5 (anxiety or attitude* or awareness or behavio#r or belief* or criticis* or doubt* or distrust or dropout* or exemption* or fear* or hesitanc* or low* or trust or mistrust or perception* or refus* or rejection or rumo#r* or intent* or controvers* or misconception* or misinformation or opposition or delay or dilemma* or objector*)) )
