# Supplementary material for: Interventions to increase vaccination in vulnerable groups: rapid overview of reviews
Source: BMC Public Health. 2024 Jun 3;24:1479. doi: 10.1186/s12889-024-18713-5 (PMC11145854; doi:10.1186/s12889-024-18713-5)
Supplement: Supplementary file 2 — Supplementary Material 2 Appendix 2: Documentation of evidence for interventions [file 12889_2024_18713_MOESM2_ESM.docx]

| **Intervention Type** | **Contributing reviews** | | **Population**  **Vaccination** | **Intervention description** | **Evidence for effect** | **Reliability assessment** |
| --- | --- | --- | --- | --- | --- | --- |
| **High intensity interventions to increase demand** | | | | | | |
| Home visits for communication or education | Glenton 2011 | | Economically disadvantaged families  Childhood vaccination | Home visits by lay health workers with or without nurses for vaccine promotion | High income findings: RR for LHW visits: 1.19 (1.09, 1.30) for LHW visits. Comparison with postal reminders showed mixed results (1 RCT). Vaccine promotion at home visits by LHW and nurses showed mixed results, mainly positive. | **Review: low risk of bias**  Content: existing GRADE judgement of moderate certainty evidence for LHW; low for LHW and nurses; differentiates high and LMIC. |
| Home visits for communication or education | Kaufman 2018 | | Low SES and adolescent mothers  Childhood vaccination | Face to face interventions including home visits by midwives or case managers for informing/educating parents | The review could not isolate the effect of the face to face component from the rest of the multicomponent intensive postnatal support interventions. | **Review: low risk of bias**  Content: existing GRADE judgement of low certainty evidence but not specific to the two RCTs in vulnerable groups. These were reasonably large but one was at high risk of bias. |
| Home visits for communication or education | Lewin 2010 | | Economically disadvantaged families  Childhood vaccination | Lay health workers encouraging using vaccination during home visits | RR for immunisation uptake: (RR 1.22 (1.10, 1.37). | **Review: low risk of bias**  Content: existing GRADE judgement of moderate certainty evidence. Very substantial overlap with Glenton (2011). Based on multiple RCTs; substantial number of participants in high income countries. |
| Home visits for communication or education | Machado 2021 | | Families with SES disadvantage  Childhood vaccination | Home visits from family specialists or outreach workers; reminders/follow-up during home visits; home visits as part of multicomponent intervention | Telephone call + home visit increased immunisation versus telephone alone (+11.8% absolute difference). Multicomponent intervention combining visits with telephone, post-cards and case management: up to date on immunisation +11% absolute difference. Combined home visits with routine visits and contact by phone/email/text: immunisation + 11-15% (p < 0.05). | **Review: low risk of bias**  Content: Reported mainly absolute differences with p values in some cases. More than one RCT assessing home visits; reasonable sample size; effect size only reported as p values. No quality assessment in review. |
| Home visits for communication or education | Thomas 2018 | | Older adults  Influenza vaccination | Door-knocking and home visits for reminders for home visits to older adults for influenza vaccination | Multiple OR with 95% CI which do not cross line of no effect. | **Review: low risk of bias**  Content: existing overall GRADE judgement of moderate certainty evidence; high certainty in some cases. |
| Home visits for communication or education | Kendrick 2000 | | Ethnic minority or low SES families  Childhood vaccination | Home visits by psychology graduates, paraprofessionals, public health or community nurses, health visitors or women/mothers from the community | Pooled analysis (9 of 11 studies) found significant benefit of home visiting; sensitivity analysis of 7 RCTs supported this (OR 1.67 (1.29, 2.15)); similar effect found for only socioeconomically disadvantaged participants (RCT+nonRCT)(OR 1.49, (1.20-1.86)). Analyses of studies using professionals only showed no difference between groups. | **Review: unclear risk of bias**  Content: Could not separate results for RCTs in disadvantaged groups. However effects were broadly similar between analyses of all RCTs and all studies in disadvantaged groups suggesting both may be similar to RCTs in disadvantaged groups. Only assessed blinded outcome assessment (mixed). |
| Home visits for communication or education | Lambert 2021 | | Migrant or refugee status individuals  Respiratory-related childhood disease in | Door-to-door visiting information campaign, combined with face masks or hand sanitizer | Single RCT evaluated impact of additional interventions (face masks/sanitiser) not impact of information visiting campaign; vaccination rates improved in all groups. | **Review: unclear risk of bias**  Content: We can’t comment on the evidence for the intervention of interest – it was not the intervention difference between the groups |
| Home visits for communication or education | Nelson 2016 | | Children with low SES in LMIC  Childhood vaccination | Home visits by undergraduate medical students who identified knowledge gaps and delivered health education messages  Home visits delivering repeated messages for those not visiting clinic after referral | Home visits increased immunisation by 19% to 38% depending on vaccine targeted. For those not visiting clinic after referral % fully immunised increased by 19% | **Review: unclear risk of bias**  Content: 3/6 RCTs low risk of bias on all domains using AHRQ criteria; other 3 were high or unclear on 1 or 2 / 5 domains. Moderate sized RCT, statistical significance not reported for all studies |
| Home visits for communication or education | Crocker-Buque 2017a | | Low SES or slum residents in LMIC  Multiple vaccinations | Home visits by undergraduate medical students who identified knowledge gaps and delivered health education messages  in LMIC. Home visits with repeated messages for clinic non-attenders | Same evidence as in Nelson 2016 | **Review: high risk of bias**  Content: as for Nelson 2016 above |
| Home visits for communication or education | Crocker-Buque 2017b | | Children and adolescents from disadvantaged, minority ethnic or urban location groups.  Multiple vaccinations | Enhanced prenatal and postnatal home visitation programme; Progressively more intense reminder/recall and outreach programme. | Enhanced prenatal & postnatal home visits showed no difference in vaccination uptake.  Progressively more intense reminder/recall and outreach programme showed increase in being up to date on vaccination (OR 1.6 (1.2, 2.1); 2^nd^ RCT also showed increased chance of being up to date with this type of intervention (+12-16%, p <0.001). | **Review: high risk of bias**  Content: Reports full effect estimates for some not all; reports both negative and positive results with equal weight. RCTs can be separated For result showing effectiveness there are 2 RCTs with reasonable sample size with same direction of effect. No quality assessment in review. |
| Home visits for communication or education | Sarnoff 1998 | | Older adults  Influenza vaccination | Home visits as single  Intervention; home visits as part of broader interventions | Result for one RCT of home visit as single intervention: OR 0.10 ( 0.70 to 1.55). For all patient-focused interventions in review pooled OR: 1.85 (1.25, 2.75). | **Review: high risk of bias**  Content: Moderate sized RCT for single intervention. No quality assessment in review. |
| Advocacy | Murray 2021 | | Older adults  Influenza vaccination | Pharmacists advocating for vaccination | One RCT assessing this: RR 1.26 (1.19 to 1.33). | **Review: low risk of bias**  Content: Large RCT with clear effect estimate and narrow CI. High risk of bias for at least one domain in all 3 RCTs (2 domains in 2/3). |
| Advocacy | Nelson 2016 | | Children with low SES in LMIC  Childhood vaccination | Community volunteers motivating acceptance of vaccination as part of the integrated child development service | Community volunteers as part of Integrated Child Development service increased vaccination by 73-74% (difference vaccines) | **Review: unclear risk of bias**  Content: intervention was part of multicomponent intervention so hard to isolate effect. 3/6 RCTs low risk of bias on all domains using AHRQ criteria; other 3 were high or unclear on 1 or 2 / 5 domains. Statistical significance not reported for all studies |
| Community partnership and outreach | Glenton 2011 | | Economically disadvantaged families  Childhood vaccination | Lay health workers leading focus group discussions with parents | LHW-led discussions increased proportion of children up to date on immunisation (RR 2.1, (1.43, 3.29)) | **Review: low risk of bias**  Content: GRADE assessment of moderate quality evidence |
| Community partnership and outreach | Machado 2021 | | Families with SES disadvantage  Childhood vaccination | Outreach to determine vaccination status and use of community volunteers as part of a multicomponent intervention  Group care visits led by nurse practitioner | There was a 26% increase in number of children up to date on immunisations (P < 0.05) | **Review: low risk of bias**  Content: 1 large and 1 moderate size RCT; effect size only reported as p values. Risk of bias not reported. The effect of individual components of the intervention is unclear. |
| Community partnership and outreach | Nelson 2016 | | Children with low SES in LMIC  Childhood vaccination | Community volunteers operating as part of integrated child development service to motivate acceptance of vaccination; community volunteers listing children for vaccination | Increases in vaccination of 73% or 74% (two different vaccinations); limited reporting of outcomes. | **Review: unclear risk of bias**  Content: moderate size RCT. Risk of bias – 3/6 total RCTs low risk of bias, 3 high or unclear on 1 or 2/5 AHRQ domains. Outcome reporting incomplete/inconsistent |
| Community partnership and outreach | Crocker-Buque 2017a  CHECK THIS | | Low SES or slum residents in LMIC  Multiple vaccinations | Community involvement as part of a multi-component campaign, including development of educational and clinic reminders | A multicomponent campaign achieved 74% coverage in previously unvaccinated population | **Review: high risk of bias**  Content: this was a cluster RCT but information about the control group was not reported; risk of bias not reported. |
| Community partnership and outreach | Vedio 2017 | | Ethnic minority or migrant/refugee status at risk of hepatitis B  Hepatitis B vaccination | Community lay-educators  Education about vaccination in ESL classes. Health service navigation and knowledge promotion | Interventions improved significantly knowledge about hepatitis B (but it remained low) but vaccination was not assessed | **Review: low risk of bias**  Content: Indirectly relevant. Statements generated included weak equivocal evidence linking knowledge to testing/vaccination. At least one large RCT;quality assessment not fully reported. |
| Community partnership and outreach | Gopalani 2022 | | First nations peoples in United States (Alaskan first nation here)  HPV vaccination | Mother-daughter dyad educational intervention | Mothers who received the intervention were more likely to initiate HPV vaccination compared to control (RR 2.6, (1.4, 4.9)) | **Review: unclear risk of bias**  Content: 1 cluster-randomised trial with limited information on study characteristics or risk of bias |
| School and other non-home based educational interventions | Nelson 2016 | | Children with low SES in LMIC  Childhood vaccination | Educational session using pictorial message delivered by child health workers. Brief educational message plus redesigned vaccination cards. | Same data as in Crocker-Buque 2017a (see there as more precision in reporting) | **Review: unclear risk of bias**  Content: single moderate sized RCT for each intervention; statistical significance reported in some instances, some only reported absolute differences. 3/6 RCTs low risk of bias on all domains (AHRQ criteria); others high or unclear on 1 or 2/5 domains. |
| School and other non-home based educational interventions | Brandt 2021 | | Adolescents in rural communities in US  HPV vaccination | School-based science education. School engagement to incorporate the information into activity and service delivery | No evidence of effect compared with brochure mailed to parents | **Review: high risk of bias**  Content: medium-sized RCTs, no risk of bias assessment, results not fully reported |
| School and other non-home based educational interventions | Crocker-Buque 2017a | | Low SES or slum residents in LMIC  Multiple vaccinations | Educational session using pictorial message delivered by child health workers  Educational message to mothers delivered by medical student | Same data as in Nelson 2016  Pictorial message increased coverage (RR 1.39, (1.06 to 1.81)). Education message from medical students increased full immunisation (p < 0.005); no change in control area (no group comparison reported). | **Review: high risk of bias**  Content: This is a more precise reporting of data in Nelson 2016 (above) but with less evaluation of study quality |
| School and other non-home based educational interventions | Crocker-Buque 2017b | | Children and adolescents from disadvantaged, minority ethnic or urban location groups.  Multiple vaccinations | Computer-based interventions in school settings/school clinics (different interventions: Girls on Guard; messaging using rhetorical question approach) | Girls on guard did not show a difference between groups in vaccine initiation  Rhetorical questioning message increased intention to be vaccinated but not vaccination. | **Review: high risk of bias**  Content: These were reasonable sized RCTs, there is no quality appraisal. Full effect estimates not always reported. |
| School and other non-home based educational interventions | Mogaka 2019 | | Low SES or ethnic minority adolescents  HPV vaccination | 10 minute educational session | The session group had higher vaccination intent (86% vs 67%; OR 3.09, (1.02, 9.30)) | **Review: high risk of bias**  Content: Indirectly relevant outcome; moderate size RCT; variable reporting of outcomes across review; included RCTs did not report blinded outcome assessment. |
| School and other non-home based educational interventions | Rani 2022 | | Minority adolescent girls or women  HPV vaccination | Mother-daughter dyad interventions for minority adolescent girls  Culturally tailored computer-delivered education in clinic for minority women | Some data duplicated from Gopalani 2022; Crocker-Buque 2017b (2 RCTs) and Brandt 2021  In the unique study a dyad intervention involving education, referral, navigations support and a brochure increased vaccination series completion (OR 2.24, (1.25-4.02)) versus brochure only. | **Review: high risk of bias**  Content: moderate sized RCT, no quality appraisal; results reported for null as well as positive findings. |
| **High intensity interventions to increase access** | | | | | | |
| Home visits for vaccination | Thomas 2018 | | Older adults  Influenza vaccination | Home visits for health checks and offer of vaccination by GP or practice nurse | OR 1.30 (1.05, 1.61) | **Review: low risk of bias**  Content: Review assessed certainty of evidence as high based on two pooled RCTs with large total size (most from one RCT). |
| Home visits for vaccination | Nelson 2016 | | Children with low SES in LMIC  Childhood vaccination | Vaccinations during home visits by undergraduate medical students as part of a multicomponent intervention (also delivered education). | There are limited between-group data presented; for four vaccinations the percentage increase between the control and intervention groups is between 23% and 38% | **Review: unclear risk of bias**  Content: One medium sized RCT; intervention is multicomponent also including delivery of education/knowledge. 3/6 RCTs low risk of bias on all domains (AHRQ criteria); others high or unclear on 1 or 2/5 domains. |
| Home visits for vaccination | Crocker-Buque 2017a | | Low SES or slum residents in LMIC  Multiple vaccinations | Vaccinations during home visits by undergraduate medical students as part of a multicomponent intervention (also delivered education). | Same data as Nelson 2016 | **Review: high risk of bias**  Content: same as Nelson 2016 |
| Additional clinics | Crocker-Buque 2017b | | Children and adolescents from disadvantaged, minority ethnic or urban location groups.  Multiple vaccinations | Additional clinics delivered as part of multicomponent intervention (also includes reminders, recalls, tracking, home visit outreach and training and prompts for providers | Greater vaccination uptake in intervention groups 9.9 vs 4.2%; p < 0.001) | **Review: high risk of bias**  Content: 2 cluster RCTs; results not fully reported; no quality assessment; intervention effect is as part of a wider complex intervention. |
| Vaccination by pharmacists | Murray 2021 | | Older adults  Influenza vaccination | In store vaccination by pharmacists plus advertising of vaccination; phone calls from pharmacist added in one group | 1 RCT of pharmacist-initiated vaccination programmes compared impact of adding phone call to intervention which included vaccination. RR 0.96 (0.84, 1.09) | **Review: low risk of bias**  Content: only one RCT in relevant population. RoB was assessed as high for at least one domain in all 3 RCTs (2 domains in 2/3). Does not assess effect of vaccination but of phone call |
| Vaccination by pharmacists | Isenor 2016 | | Older adults, low SES  Influenza, herpes zoster or multiple vaccination | Vaccination by pharmacists; cointerventions present (invitations, personal health record, needs assessment) | Cluster RCT: 80.1% (invitations) vaccinated vs 56.9% . Personal health record in conjunction with pharmacist intervention: 13.2% vs 5% and 5% vs 1.8% vaccinated; small RCT – intervention protocol 18x more likely to be current on immunisations compared to control. | **Review: low risk of bias**  Content: 2 large RCTs, 1 small, all at high risk of bias. Results not fully reported; cointerventions present but controlled for in one but not both |
| Vaccination by pharmacists | Sarnoff 1998 | | Older adults  Influenza vaccination | Vaccination by pharmacists; free vaccination and patient reminder also included | One RCT; OR 4.03 (3.54, 4.58) | **Review: high risk of bias**  Content: One large RCT, intervention included other elements; no quality assessment |
| Vaccination involving lay or community healthcare workers | Glenton 2011 | | Economically disadvantaged families  Childhood vaccination | Vaccination by lay healthcare workers | One RCT at high risk of bias reported an increase in number of children up to date on measles vaccination but unclear if result was statistically significant. | **Review: low risk of bias**  Content: Review assessed this evidence as very low certainty. Bsaed on an RCT excluded from a Cochrane review for low quality |
| Vaccination involving lay or community healthcare workers | Nelson 2016 | | Children with low SES in LMIC  Childhood vaccination | Vaccination teams assisted by community healthcare workers who managed vaccination adverse events. Part of integrated child development programme (listing and motivating for vaccination also included) | Vaccinations increased by 73 or 74% (3 different vaccines) | **Review: unclear risk of bias**  **Content:** Large RCT but only small control group (126 vs 1715 intervention). Results not fully reported. 3/6 RCTs low risk of bias on all domains (AHRQ criteria); others high or unclear on 1 or 2/5 domains. |
| **High intensity provider-focused interventions** | | | | | | |
| Facilitators working with healthcare practices | Thomas 2018 | | Older adults  Influenza vaccination | Facilitators to support multiple goals including influenza vaccination. Two used multiple strategies including practice visits, baseline audit, feedback, consensus building, follow-up. One combined visits with educational materials for professionals and patients | Trials all showed benefit with 95% CI which did not cross line of no effect. | **Review: low risk of bias**  **Content: 3** cluster RCTs, total of over 2000 patients. High heterogeneity but consistent direction of effect. The review graded the evidence as moderate certainty based on a risk of incomplete data in one trial. |
| Education or training for providers | Machado 2021 | | Families with SES disadvantage  Childhood vaccination | Educational interventions for physicians | This was not reported as one of the effective interventions or strongly rated studies; no further information | **Review: low risk of bias**  Content: very limited information |
| Education or training for providers | Brandt 2021 | | Adolescents in rural communities in US  HPV vaccination | Provider training: announcement vs conversational recommendation vs control; education session plus prompts and follow-up calls | Provider training interventions increased vaccination initiation by 5.4%.  Education session did not increase vaccination. | **Review: high risk of bias**  Content: Large cluster RCT for training interventions; smaller cluster RCT for education session. Results not fully reported; no quality assessment. |
| Education or training for providers | Crocker-Buque 2017b | | Children and adolescents from disadvantaged, minority ethnic or urban location groups.  Multiple vaccinations | Healthcare worker training as part of multicomponent intervention (includes additional clinics, reminders, recalls, tracking, home visit outreach and prompts for providers) | Greater vaccination uptake in intervention groups 9.9 vs 4.2%; p < 0.001) | **Review: high risk of bias**  Content: 2 cluster RCTs; results not fully reported; no quality assessment; intervention effect is as part of a wider complex intervention. |
|  | | **Medium intensity interventions to increase demand** | | | | |
| Phone calls to communicate or educate | Lewin 2010 | | Economically disadvantaged families; ethnic minority groups  Childhood vaccination | Phone calls from lay healthcare workers to encourage vaccination; part of multicomponent intervention (postcards, home visits to non-responders) | LHW increase immunisation uptake RR 1.22 (1.10 to 1.37) | **Review: low risk of bias**  Content: 1 large RCT, low risk of bias; overall judgement for LHW interventions moderate certainty |
| Phone calls to communicate or educate | Glenton 2011 | | Economically disadvantaged families  Childhood vaccination | Phone calls from lay healthcare workers to encourage vaccination; part of multicomponent intervention (postcards, home visits to non-responders) | Same evidence as Lewin 2010 | **Review: low risk of bias**  Content: Same evidence as Lewin 2010 |
| Phone calls to communicate or educate | Thomas 2018 | | Older adults  Influenza vaccination | Telephone calls to remind people to book or attend appointments, as either a standalone intervention or a multicomponent/staged approach | Review found personalised phone calls were effective. Reminder and recall interventions often grouped calls with postal reminders; both simple and personalised reminders were effective, adding a leaflet/postcard to a postal reminder was also effective. Multiple ORs reported in review. | **Review: low risk of bias**  Content: GRADE assessment of low to moderate certainty in review; multiple RCTs showing consistent evidence. Possibly indirect for minoritized groups (elderly). |
| Phone calls to communicate or educate | Machado 2021 | | Families with SES disadvantage  Childhood vaccination | Phone calls as part of a multicomponent intervention (tracking system, phone calls and postcards); computer generated telephone reminder | Multicomponent interventions improved immunisation rates by 17-25% (p < 0.05); computer-generated reminder improved on-time immunisation by 11.6%. | **Review: low risk of bias**  Content: Multiple RCTs (varying size) showing consistent impact for tracking and reminders including phone calls, impact of phone calls in these cannot be isolated. Only p values reported; no quality assessment. |
| Phone calls to communicate or educate | Brandt 2021 | | Adolescents in rural communities in US  HPV vaccination | Educational phone calls as part of wider intervention | Intervention increased 1st dose (not fully reported) | **Review: high risk of bias**  Content: single RCT, moderate size;  not fully reported; no quality assessment |
| Phone calls to communicate or educate | Crocker-Buque 2017b | | Children and adolescents from disadvantaged, minority ethnic or urban location groups.  Multiple vaccinations | Targeted telephone calls  Telephone reminders to parents and/or adolescents; phone calls as part of a progressively more intense reminder/recall and outreach programme | Targeted calls to adolescents and parents more effective than parents alone OR 2.27 (1.00, 5.18)  Progressively more intense reminder/recall programme showed significant increase in up to date vaccination (OR 1.6 (1.2 to 2.1); (12-16% more likely, p < 0.001)(2 different RCTs) | **Review: high risk of bias**  Content: progressive reminders: 2 reasonably large RCTs with same effect direction; no quality assessment; only some studies reported with full effect estimates; |
| Phone calls to communicate or educate | Sarnoff 1998 | | Older adults  Influenza vaccination | Telephone reminders to book or attend appointments | Reported as pooled result for six RCTs of patient-focused interventions: OR 1.85 (1.25, 2.75); evidence for mixed patient/provider focused interventions included non-RCTs. | **Review: high risk of bias**  **Content:** 6 patient-focused RCTs showed consistent direction of effect, not clear how many included telephone call; all 6 involved reminders; at least one used phone calls. No quality assessment; effect of phone calls cannot be isolated. |
| Printed material in person | Nelson 2016 | | Children with low SES in LMIC  Childhood vaccination | Redesigned immunisation cards  Pictorial information cards | Redesigned cards increased immunisation by 19% vs control; cards + education increased by 10% vs control. Pictorial information cards in home visit increased immunisation by 20% vs control. | **Review: unclear risk of bias**  Content: Immunisation cards: larger RCT; Pictorial information:  moderate size RCT; only absolute differences reported. 3/6 RCTs low risk of bias on all 5 domains using AHRQ criteria; other 3 were high/unclear on 1 or 2 domains. |
| Printed material in person | Crocker-Buque 2017a | | Low SES or slum residents in LMIC  Multiple vaccinations | Pictorial information cards | Same data as in Nelson 2016 | **Review: high risk of bias**  Content: Same data as in Nelson 2016 |
| Printed material in person | Callahan 2021 | | Pregnant women from ethnic minority groups  Influenza vaccination | Pamphlet of information plus a verbal message of benefits of vaccination | Pamphlet vs pamphlet + verbalised benefit vs control: both intervention groups had higher vaccination rates vs control (p < 0.01); 13% higher in combined intervention groups but significance not reported | **Review: high risk of bias**  Content: moderate sized RCT; clear consistent reporting but no quality assessment |
| Printed material in person | Mogaka 2019 | | Low SES or ethnic minority adolescents  HPV vaccination | Information sheet/flyer plus free t-shirt | More (43.3% vs 31.9%) completed 3 dose schedule (p = 0.03) | **Review high risk of bias**  Content: moderate sized RCT; incomplete outcome reporting; some quality assessment – RCTs did not use observer blinding. |
| **Medium intensity interventions to increase access** | | | | | | |
| Increasing visibility of vaccinator | Nelson 2016 | | Children with low SES in LMIC  Childhood vaccination | Putting immunisation desk at front of clinic versus referring to vaccinator in separate room | Vaccinator at front of clinic: 61% immunised on day vs 66% for referral | **Review: unclear risk of bias**  Content: relatively small sized RCT comparing 2 interventions; RoB low for this study; incomplete reporting with only absolute values reported |
| Using routine visits to vaccinate | Machado 2021 | | Families with SES disadvantage  Childhood vaccination | Using routine clinic/ healthcare provider visits to vaccinate. Part of multicomponent intervention (outreach/ home visits/ reminders; feedback to providers) | Increased percentage of up to date immunisation coverage by 3-21% (p < 0.05) | **Review: low risk of bias**  Content: 1 large RCT; effect size only reported as p values. No quality assessment. Cannot isolate effect of this component of intervention. |
| Using routine visits to vaccinate | Sarnoff 1998 | | Older adults  Influenza vaccination | Instructions to nurses to vaccinate vulnerable patients | Only pooled results reported for all provider-focused interventions (as subgroup of whole analysis); only one was an RCT. OR 2.06 (1.70 to 2.48). Mixed patient/ provider focused interventions assessed by both RCT and non-RCTs; OR 2.50 (1.75, 3.58); (OR for RCTs in all interventions 1.96 (1.54, 2.49)) | **Review: high risk of bias**  Content: Only one of contributory studies for provider only interventions was an RCT; mixed evidence for combined patient/provider focused interventions. Indirectly relevant; no quality assessment. |
| Using routine visits to vaccinate | Odone 2015 | | Ethnic minority groups  Childhood vaccination | Provider-level standing orders to vaccinate vs physician reminder vs control | Vaccination of standing order group patients ranged from 42-73%; physician reminder group patients from 15-59.7%; these percentages “were much higher than in control groups with no intervention” | **Review: unclear risk of bias**  Content: Results combined RCT and non RCT evidence; effects not fully reported. Downs & Black assessment, reasonable score. Large single RCT |
| **Medium intensity provider-focused interventions** | | | | | | |
| Case management | Machado 2021 | | Families with SES disadvantage  Childhood vaccination | Case management  Tracking, triage, assessment and flagging of vaccination status as part of multi-component intervention (e.g. using clinic visits to vaccinate, home visits) | Case management as main intervention (but main patient component home visit); increased proportion up to date by 13.2% (p < 0.05). Multicomponent intervention including case management increased up to date on immunisation by 11%. | **Review: low risk of bias**  Content: 1 large RCT; effect size only reported as p values. Second RCT only reported absolute differences. Cannot isolate effect of this component of intervention. No quality assessment. |
| **Low intensity interventions to increase demand** | | | | | | |
| Text messages to communicate or educate | Odone 2015 | | Ethnic minority groups  Childhood vaccination | Text messages to parents of babies due for immunisation  Text messages to parents of children/adolescents due Hib/DTP /meningococcal vaccination  Compared to standard mail reminder | Increased percentage of due immunisations received (only absolute numbers reported) at all timepoint; Increased vaccination at each age assessed and attendance at special immunization recall sessions (all statistically significant p < 0.005 where reported) | Review: low risk of bias  Content: 4 RCTs – small to very large sample sizes. Generally consistent set of results with one inconsistent one clearly reported. Quality assessment Downs & Black; RCTs medium to high scores with 1 reported exception. |
| Text messages to communicate or educate | Machado 2021 | | Families with SES disadvantage  Childhood vaccination | Texts for scheduling and reminding of appointments vs reminder only | Increased MMR vaccine coverage by 6% | **Review: low risk of bias**  Content: Large RCT; Partial reporting of outcome data; no quality assessment. |
| Text messages to communicate or educate | Brandt 2021 | | Adolescents in rural communities in US  HPV vaccination | Text reminders | Results not clearly reported | **Review: high risk of bias**  Content: results unclear; also unclear if population in this RCT is relevant. No quality assessment. |
| Text messages to communicate or educate | Callahan 2021 | | Pregnant women from ethnic minority groups  Influenza vaccination | 12 weekly text messages on benefits of vaccination | 31% of control group and 33% of intervention group were vaccinated (p = 0.88). | **Review: high risk of bias**  Content: 1 moderate sized RCT; clear reporting; no quality assessment. |
| Text messages to communicate or educate | Crocker-Buque 2017b | | Children and adolescents from disadvantaged, minority ethnic or urban location groups.  Multiple vaccinations | Text message reminders to schedule appointment, reminders of appointment details; Series of text messages (and letter with financial incentive);  Information as part of multi-component campaign  Educational vs conventional texts; interactive vs educational texts;5 weekly community-developed texts series | Texts as part of progressively intense programme: increase in up to date on vaccination (OR 1.6 (1.2 to 2.1)); (12-16% more likely, p < 0.001).Text messages monitoring of immunisation – 84.4% vs 80.7% in control (NS). Text messages vs control only increased uptake in children without booked appointment RR 1.11 (1.00, 1.24). Educational text messages more likely to get 2^nd^ influenza shot (p = 0.003) vs conventional text messages/ postal reminders. Interactive texts increased proportion vaccinated RR 1.09 (1.00 to 1.19). Text series increased proportion vaccinated (RR 1.09 (1.04 to 1.15)) | **Review: high risk of bias**  Content: Full effect estimates inconsistently reported; varying sized of RCTs. No quality assessment. |
| Email reminders or online messaging | Lott 2020 | | Minority groups (ethnic or LGBTQ) of young people  HPV vaccination | Population-targeted individually tailored online reminders  7 electronic messages with health education and reminders | Individually tailored online reminders improved initiation within 7 months (Or 2.34 (1.18-4.67)); not clearly completion in 7 months OR 4.24 (0.87 to 20.66). 7 electronic messages: effect on series completion within 7 months varied among minority groups | **Review: Unclear risk of bias**  Content: single RCT for each intervention; risk of bias assessment: at least one high RoB domain for all except one RCT; some trials had 2. Clear reporting of results. |
| Email reminders or online messaging | Machado 2021 | | Families with SES disadvantage  Childhood vaccination | Use of email as well as phone calls and text messages in multicomponent intervention (also home visits) | Increased immunisation overage by 11-15% (p < 0.05) | Review: low risk of bias  Content: 1 medium sized RCT; no quality assessment in review; cannot isolate effect of email component in intervention. |
| Email reminders or online messaging | Brandt 2021 | | Adolescents in rural communities in US  HPV vaccination | Email reminders as well as text messages to parents | It’s not clear whether this targeted a vulnerable group; no effect found. | **Review: high risk of bias**  Content: May not be directly relevant; inconsistent and incomplete reporting of outcomes; no quality assessment. |
| Video messaging | Mogaka 2019 | | Low SES or ethnic minority adolescents  HPV vaccination | Educational video plus information and T-shirt | More (43.3% vs 31.9%) completed 3 dose schedule (p = 0.03) | **Review: low risk of bias**  Content: Limited outcome reporting; moderate sized RCT; some quality assessment – RCTs did not use observer blinding. |
| Video messaging | Lott 2020 | | Minority groups (ethnic or LGBTQ) of young people  HPV vaccination | Bilingual video material; Culturally appropriate storytelling intervention; Multicomponent intervention with culturally appropriate video and keychain reminder | No effect of video plus keychain in multicomponent intervention.  Educational video in English/Khmer made no difference to vaccine initiation but increased intention to receive. Culturally appropriate storytelling did not change outcomes including scheduling first vaccine. | **Review: Unclear risk of bias**  Content: single RCT for each intervention; risk of bias assessment: at least one high RoB domain for all except one RCT; some trials had 2. Clear reporting of results. Some studies very small. |
| Video messaging | Brandt 2021 | | Adolescents in rural communities in US  HPV vaccination | Video by mailed DVD combined with phonecall or part of multicomponent intervention; printed material also sent. | DVD combined with phone call for newly vaccinated women increased completion rate; DVD for adolescents and parents part of multicomponent intervention (7.7% vs 3.2% vaccinated) | **Review: high risk of bias**  Content: Medium sized RCTs in each case; inconsistent and incomplete reporting of outcomes; no quality assessment. |
| Video messaging | Callahan 2021 | | Pregnant women from ethnic minority groups  Influenza vaccination | Active or cognitive video messaging. Loss-framed vs game-framed messages  Short video (5 minutes  Ipads with patient-centred tutorial plus written/other materials | 3 RCTs: none found any statistically significant difference between the groups (loss vs gain-framing; active vs cognitive videos; short video on immunization vs handwashing video) | **Review: high risk of bias**  Content: small -medium sized RCTs comparing two different interventions in each case, no usual care groups. Clear consistent reporting bur no quality assessment. |
| Video messaging | Rani 2022 | | Minority adolescent girls or women  HPV vaccination | Video by mailed DVD | Same data as in Brandt 2021 | **Review: high risk of bias**  Content: same data as in Brandt 2021 |
| Printed material by post: letters or postcards or other forms | Lewin 2010 | | Economically disadvantaged families  Childhood vaccination | Postcards from lay health workers to encourage vaccination; part of multicomponent intervention (phone calls, home visits to non-responders) | LHW increase immunisation uptake RR 1.22 (1.10 to 1.37) | **Review: low risk of bias**  Content: 1 large RCT, low risk of bias; overall judgement for LHW interventions moderate certainty |
| Printed material by post: letters or postcards or other forms | Machado 2021 | | Families with SES disadvantage  Childhood vaccination | Postcards  Personalised letters  Part of multicomponent interventions | Patient-specific/visit-specific letter/postcard increased those immunised by 2.5-5.9% (p < 0.05);  Tracking system with postcard/telephone reminders increased completion rates by 17-25% (p < 0.05) | **Review: low risk of bias**  Content: Multiple small to large size RCTs showing consistent impact for tracking and reminders including postal/texts. Only p values reported. No quality assessment. |
| Printed material by post: letters or postcards or other forms | Thomas 2018 | | Older adults  Influenza vaccination | Postcards, leaflets and other print media | Postcards increased vaccination in 11/17 RCTs; a reminder by either a tailored letter, postcard or phone call increased vaccination in 12/16 RCTs. Customised letters or phone calls increased vaccination in 2/4 RCTs compared to form letters.  Letter plus leaflet or postcard compared to letter OR 1.11 (1.07, 1.15) | **Review: low risk of bias**  Comment: Large numbers of RCTs but no pooling due to high statistical heterogeneity. Review graded this evidence as high certainty (taking this into account as well as RoB, imprecision etc). Phone calls in some interventions mean some impact may not be from postal material. Possibly indirect for minoritized groups. |
| Printed material by post: letters or postcards or other forms | Brandt 2021 | | Adolescents in rural communities in US  HPV vaccination | Educational brochure as part of multicomponent intervention; printed material in combination with DVD by mail; Brochure as control for school science education | Brochure plus DVD: 7.7% vs 3.2% of control participants received 1^st^ dose within 3 months’  control for science education showed no difference between interventions | **Review: high risk of bias**  Content: modest size RCT, only absolute numbers reported; no quality assessment |
| Printed material by post: letters or postcards or other forms | Crocker-Buque 2017b | | Children and adolescents from disadvantaged, minority ethnic or urban location groups.  Multiple vaccinations | Universal language-appropriate reminder postcards (first stage of escalating intervention)  Promotional materials  Patient reminder/recall  Postal reminder or recall notice | Postal reminders increased vaccination up to date at 19 months (p < 0.001) but not 7 or 12 months; other reminders delivered as part of multicomponent or escalating intensity interventions: intervention groups showed bigger increases in vaccination (p < 0.001) | **Review: high risk of bias**  Content: Trial sizes varied; full effect estimates not reported; no quality assessment. |
| Printed material by post: letters or postcards or other forms | Lott 2020 | | Minority groups (ethnic or LGBTQ) of young people  HPV vaccination | Vaccine reminder letter in English/Spanish every 3 months | Increased completion within 12 months in each of 3 minority ethnic groups (p < 0.01) | **Review: unclear risk of bias**  Content: single RCT for each intervention; risk of bias assessment: at least one high RoB domain for all except one RCT; some trials had 2. Clear reporting of results. |
| Printed material by post: letters or postcards or other forms | Mohammed 2021 | | First nations people  Influenza vaccination in children | Pamphlets designed following recommendations for health information resources for Aboriginal communities in Australia versus personalised letters | Pamphlets did not improve influenza vaccination rates in children but personalised letters increased vaccination rates by 34%. | **Review: high risk of bias**  Content: single RCT, limited information, no assessment of risk of bias |
| Printed material by post: letters or postcards or other forms | Sarnoff 1998 | | Older adults  Influenza vaccination | Mailed reminders | Only pooled results reported for all provider-focused interventions (as subgroup of whole analysis); only one was an RCT. OR 2.06 (1.70 to 2.48). Mixed patient/ provider focused interventions assessed by both RCT and non-RCTs; OR 2.50 (1.75, 3.58); (OR for RCTs in all interventions 1.96 (1.54, 2.49)) | **Review: high risk of bias**  Content: Only one of contributory studies for provider only interventions was an RCT; mixed evidence for combined patient/provider focused interventions. Indirectly relevant; no quality assessment. |
| Mass media | Vedio 2017 | | Ethnic minority or migrant/refugee status at risk of hepatitis B  Hepatitis B vaccination | Mass media as part of a multicomponent intervention (community elements as well including physician engagement) | Increased knowledge and awareness as well as vaccination rates but these remained low and it’s not clear whether the impact on vaccination was significant. | **Review: low risk of bias**  Content: Results not clearly reported; quality assessment undertaken but not fully reported; mostly focused on indirectly relevant outcomes. |
| Mass media | Mogaka 2019 | | Low SES or ethnic minority adolescents  HPV vaccination | Radionovella in Spanish (5 minute episode) | “increase in likeliness of daughters receiving HPV vaccine” | **Review: high risk of bias**  Content: very limited reporting of results; small RCT; included RCTs did not report blinded outcome assessment. |
| **Low intensity provider-focused interventions** | | | | | | |
| Reminder/recall systems | Odone 2015 | | Ethnic minority groups  Childhood vaccination | Computerised reminders to vaccinate | Vaccination rates reported grouped with non RCT results: Standing order group patients receiving vaccine ranged from 42-73%; physician reminder group patients from 15-59.7%; these percentages “were much higher than in control groups with no intervention” | **Review: unclear risk of bias**  Content: Grouped RCT and non RCT evidence; effects not fully reported. Downs & Black assessment, reasonable score. Large single RCT |
| Reminder/recall systems | Crocker-Buque 2017b | | Children and adolescents from disadvantaged, minority ethnic or urban location groups.  Multiple vaccinations | Centralised vs practice based reminder/recall systems | Centralised reminder/recall systems increased chance of children being up to date on vaccination 2 RCTs: RR 1.23 (1.10, 1.37)); RR 1.31 (1.16 to 1.48) | **Review: high risk of bias**  Content: 2 large RCTs; no quality assessment; results fully reported. |
| Electronic health records | Balzarini 2020 | | Older adults  Multiple vaccines; herpes zoster here | Use of personal electronic health records. Used in conjunction with use of digital communication features (educational, reminder and scheduling messages) | Significantly higher vaccination rates with messages compared to people without the message or without the electronic health record (with or without postal messages) (p = 0.0001) | **Review: low risk of bias**  Single RCT with low risk of bias but results not fully reported. Not assessing the EHCR itself but its use to send messages. |
| Vaccine champion | Callahan 2021 | | Pregnant women from ethnic minority groups  Influenza vaccination | Vaccine champion with provider-patient talking points plus brochures, posters, lapel pins, ipads loaded with patient-centred tutorial. | No difference between groups; study-adjusted RD 3.6% (-4.0, 11.2) for intervention practices. | **Review: high risk of bias**  Content: moderate sized RCT; no quality assessment; clear reporting. |
| Opt-in consent form | Callahan 2021 | | Pregnant women from ethnic minority groups  Influenza vaccination | Comparison of opt-in and opt-out consents | No difference between groups but both showed high rates of vaccination (83% vs 84%; p = 0.87) | **Review: high risk of bias**  Content: Reasonable size RCT; no quality assessment; effect estimate not fully reported |
| Vaccine champion | Crocker-Buque 2017b | | Children and adolescents from disadvantaged, minority ethnic or urban location groups.  Multiple vaccinations | Delivered as part of a multicomponent intervention (also includes additional clinics, reminders, recalls, tracking, home visit outreach and training for providers) | Greater vaccination uptake in intervention groups 9.9 vs 4.2%; p < 0.001) | **Review: high risk of bias**  Content: 2 cluster RCTs; results not fully reported; no quality assessment; intervention effect is as part of a wider complex intervention. |
| Prompts/feedback to healthcare workers | Machado 2021 | | Families with SES disadvantage  Childhood vaccination | Part of a multicomponent intervention (with case management, outreach/home visits/vaccination at routine visits and reminders) | Prompts increased vaccination by between 3-21%; feedback by 14-16% | **Review: low risk of bias**  Content: reasonable size RCTs; partial outcome reporting without statistical significance; no quality assessment |
| Prompts/feedback to healthcare workers | Thomas 2018 | | Older adults  Influenza vaccination | Reminders; posters in clinic with vaccination rates; educational reminders, academic detailing, peer comparisons vs educational material or chart review and feedback +/- benchmarking; payments to physicians | Reminder: 3/4 RCTs showed clear benefit; reminder for all vs ½ participants OR 2.47 (1.53, 3.99). Posters OR 2.03 (1.86, 2.22) Reminders, detailing and peer comparisons OR 1.13(0.80, 1.58); benchmarking OR3.43 (2.37, 4.97);payments OR2.22 (1.77, 2.77) | **Review: low risk of bias**  Content: review assessed these as high certainty except for educational reminders and feedback which were moderate certainty; these were mostly based on large or very large numbers of participants in between 1 and 4 RCTs |
| Prompts to healthcare workers | Sarnoff 1998 | | Older adults  Influenza vaccination | Office-based reminder systems/prompts | Only pooled results reported for all provider-focused interventions (as subgroup of whole analysis); only one was an RCT. OR 2.06 (1.70 to 2.48). Mixed patient/ provider focused interventions assessed by both RCT and non-RCTs; OR 2.50 (1.75, 3.58); (OR for RCTs in all interventions 1.96 (1.54, 2.49)) | **Review: high risk of bias**  Content: Only one of contributory studies for provider only interventions was an RCT; mixed evidence for combined patient/provider focused interventions. Indirectly relevant; no quality assessment. |
